# Chromosome‐level haplotype‐resolved genome assembly provides insights into the highly heterozygous genome of Italian ryegrass (*Lolium multiflorum* Lam.)

**DOI:** 10.1002/tpg2.70079

**Published:** 2025-08-25

**Authors:** Yutang Chen, Jenny Kiesbauer, Dario Copetti, Daniel Frei, Jürg E. Frey, Christoph Grieder, Roland Kölliker, Bruno Studer

**Affiliations:** ^1^ Molecular Plant Breeding, Institute of Agricultural Sciences ETH Zurich Zurich Switzerland; ^2^ Fodder Plant Breeding Division of Plant Breeding, Agroscope Zurich Switzerland; ^3^ Arizona Genomics Institute, School of Plant Sciences University of Arizona Tucson Arizona USA; ^4^ Department of Methods Development and Analytics Agroscope Wädenswil Switzerland; ^5^ Institute of Medical Virology University of Zurich Zurich Switzerland

## Abstract

Italian ryegrass (*Lolium multiflorum* Lam.) is an important forage grass, providing a major source of feed for ruminants in temperate regions. Due to its highly heterozygous and repeat‐rich genome, high‐quality chromosome‐level genome assemblies are scarce for Italian ryegrass. Here, we sequenced the genome of a genotype from the Italian ryegrass cultivar ‘Rabiosa’ (hereafter referred to as Rabiosa), and we obtained Oxford Nanopore Technologies long reads (∼60‐fold coverage), Illumina short reads (∼85‐fold coverage), and high‐throughput chromosome conformation capture data (∼60‐fold coverage). With Rabiosa as one of the parents, we constructed an F_1_ population consisting of 304 individuals, which were genotyped by reduced representation sequencing for linkage map construction and quantitative trait locus (QTL) analysis. Using whole‐genome sequencing data of Rabiosa and the genetic linkage map, we first generated a chromosome‐level unphased haploid assembly (scaffold N50 of 338.75 Mb, total Benchmarking Universal Single‐Copy Orthologs [BUSCO] score of 94.60%). Then, based on the unphased assembly and a reference‐based phasing approach, we generated a chromosome‐level haplotype‐resolved assembly containing both haplotypes (scaffold N50 of ∼250 Mb and total BUSCO score of ∼90% for each haplome). Between the two haplotypes of Rabiosa, we observed a highly collinear gene order at the chromosome level and a high sequence variation at the local level. With a graph‐based reference built from the unphased and the haplotype‐resolved assemblies of Rabiosa, we conducted a QTL analysis, and two QTL significantly associated with stem rust resistance were detected. The genome assemblies of Rabiosa will serve as invaluable genomic resources to facilitate genomic applications in forage grass research and breeding.

## Core Ideas

- The first chromosome‐level haplotype‐resolved assembly for highly heterozygous Italian ryegrass was generated.
- A customized haplotig purging pipeline and a reference‐based phasing pipeline were established.
- Highly consistent gene order but low local sequence identity was observed between the two haplotypes.
- Two quantitative trait loci (QTL) significantly associated with stem rust resistance were detected using a graph‐based reference.

## Plain Language Summary

Italian ryegrass is an important forage crop used to feed livestock in temperate regions worldwide. However, the nature and complexity of its genome limit the application of genomics‐based breeding strategies. Using a combination of advanced sequencing technologies, including long and short DNA reads and long‐range linkage information, we provide the first genome assembly of Italian ryegrass that is separated for the two sets of chromosomes inherited from each parent (i.e., haplotype‐resolved). We validated the usefulness of our genome assemblies for genetic analyses and identified two genome regions that are associated with resistance to stem rust, an major disease for Italian ryegrass. Our genome assemblies provide a valuable resource for future research and breeding efforts aimed at improving Italian ryegrass and other forage crops.

**Abbreviations**
BLUE: best linear unbiased estimator
BUSCO: Benchmarking Universal Single‐Copy Orthologs
CIM: composite interval mapping
GBS: genotyping‐by‐sequencing
GWAS: genome‐wide association study
Hi‐C: high‐throughput chromosome conformation capture
INDEL: insertion and deletion
LG: linkage group
LOD: logarithm of odds
ONT: Oxford Nanopore Technologies
PacBio HiFi: Pacific Biosciences' high‐fidelity sequencing technology
PAV: presence and absence variation
PGGB: pangenome graph builder
QTL: quantitative trait locus/loci
SNP: single nucleotide polymorphism
TE: transposable element
WGA: whole‐genome alignment
WGS: whole‐genome sequencing

## 1 INTRODUCTION

Italian ryegrass (*Lolium multiflorum* Lam.) is one of the most important forage grasses widely grown in temperate regions across the world, providing highly digestible and palatable feed for livestock (Boller et al., 2010). It is a diploid, outcrossing species containing seven pairs of chromosomes (2*n* = 2*x* = 14) with a very high level of heterozygosity (Copetti et al., 2021). Its genome size is estimated to be around 2.5 Gb (Copetti et al., 2021), of which 70% are repetitive sequences (Zwyrtková et al., 2020). The relatively large genome size, together with the high degree of repetitiveness and heterozygosity, makes genome assembly of this species very challenging. As a result, so far only two draft genome assemblies have been reported with one being highly fragmented (Knorst et al., 2019) and the other being haplotype‐redundant (Copetti et al., 2021). Thanks to recent advances in long‐read sequencing technologies, a chromosome‐level haploid assembly of Italian ryegrass has been generated with Pacific Biosciences’ high‐fidelity sequencing technology (PacBio HiFi) long reads (Brunharo et al., 2025). While this haploid assembly showed a high quality in terms of contiguity, completeness, and correctness, it may miss some important alleles of its heterozygous diploid genotype. Biased results might be obtained for downstream genomic applications when relying on a haploid reference. Therefore, to further advance forage grass research and breeding with the rapidly advancing long‐read sequencing technologies, haplotype‐resolved diploid assemblies containing both haplotypes are now needed.

Traditionally, when de novo assembling a heterozygous diploid genome, the assembly algorithm randomly selects only one of the two alleles to be present in the output sequence, leading to a mosaic haploid assembly with alleles among loci randomly alternating between haplotypes (Chin et al., 2016; Garg et al., 2021). With half of the genetic variation missing, a mosaic haploid assembly is never the best representation of a highly heterozygous diploid genome. Ideally, a heterozygous diploid genome should always be represented as a haplotype‐resolved assembly where alleles from both homologous chromosomes are not only present but also correctly combined or phased (Cheng et al., 2021; Garg et al., 2021; Koren et al., 2018; Llamas et al., 2021). To generate haplotype‐resolved assemblies with long‐read sequencing technologies such as Oxford Nanopore Technologies (ONT) or PacBio HiFi, so far, three approaches have been developed and adopted: bin‐then‐assemble, such as trio‐binning (Koren et al., 2018) and reference‐based phasing (Garg et al., 2021; Porubsky et al., 2021); assemble‐then‐bin, such as ALLHiC (Guk et al., 2022), HapHiC (Zeng et al., 2024), and phasing with pollen or pedigree sequencing data (Bao et al., 2022; Serra Mari et al., 2024; Sun et al., 2022; Q. Zhou et al., 2020); and graph‐based phasing, such as Hifiasm (Cheng et al., 2021) and Verkko (Rautiainen et al., 2023). These methods have generated promising haplotype‐resolved assemblies, suggesting that it is now feasible to generate haplotype‐resolved assemblies also for highly heterozygous grass species such as Italian ryegrass.

Recently, a graph‐based human pangenome reference was published (Liao et al., 2023), showing the advantage of overcoming reference bias using a pangenome reference instead of a single reference. In plant sciences, more and more pangenomic studies for grass species such as wheat (*Triticum aestivum* L., Walkowiak et al., 2020), barley (*Hordeum vulgare* L., Jayakodi et al., 2020), maize (*Zea mays* L., Gui et al., 2022), rice (*Oryza sativa* L., Shang et al., 2022), and various species of millet (*Panicum miliaceum* L., J. Chen et al., 2023; *Setaria italica* (L.) P. Beauv., He et al., 2023; *Pennisetum glaucum* (L.) R. Br., Yan et al., 2023) have also emerged. All these studies illustrate the transition from single‐reference‐dependent genomics to pangenomic approaches. Given its high level of genetic diversity and its importance as a source of forage, pangenomic resources for Italian ryegrass should be generated. Moreover, to pave the way for future pangenomic studies for *Lolium* species, methods such as pangenome graph construction, haplotype‐aware read mapping, and variant genotyping with the graph‐based reference could be tested with Italian ryegrass.

In this work, we first aimed at generating a haplotype‐resolved genome assembly for a highly heterozygous Italian ryegrass genotype using ONT long reads with high‐throughput chromosome conformation capture (Hi‐C) data and a genetic linkage map. Then, based on the resulting haplomes, we aimed at understanding the high level of heterozygosity in the genome and addressing how high heterozygosity might affect genome assembly and phasing. Finally, to explore the potential of current pangenomic methods, we aimed at building a graph‐based reference based on the haplotype‐resolved assembly and conducting quantitative trait locus (QTL) analysis for resistance to stem rust with the graph‐based reference.

## 2 METHODS

### 2.1 Plant material

A genotype (M.02402/16) of the Swiss Italian ryegrass cultivar ‘Rabiosa’ (Agroscope) was selected for whole‐genome sequencing (WGS). This genotype, referred to as Rabiosa, was crossed with a genotype (M.2002/18) of another Italian ryegrass cultivar ‘Sikem’ (DLF Seeds A/S), resulting in a bi‐parental population of 304 F_1_ individuals. Both Rabiosa and Sikem are highly heterozygous genotypes.

### 2.2 Plant material for QTL analysis

In total, 304 F_1_ seeds, collected from both Rabiosa and Sikem spikes, were germinated for 2 days in Petri dishes. The seedlings were transplanted to the greenhouse and were grown there for approximately 4 weeks. Two clonal replicates of a subset of 135 plants (133 F_1_ individuals and the two parents) were planted in autumn 2018 in a first environment at Zurich (47.427° N, 8.516° E) using an alpha design. Because of severe drought, only one replicate of each plant could be phenotyped in 2019. After phenotyping in 2019, the plants were dug out and divided into three clonal replicates and planted in a second environment at Zurich (47.427° N, 8.516° E) using an alpha design. After phenotyping in 2020, these plants were again transplanted to a third environment at Zurich (47.427° N, 8.516° E) using an alpha design and again phenotyped in three clonal replicates in 2021.

**Core Ideas**

- The first chromosome‐level haplotype‐resolved assembly for highly heterozygous Italian ryegrass was generated.
- A customized haplotig purging pipeline and a reference‐based phasing pipeline were established.
- Highly consistent gene order but low local sequence identity was observed between the two haplotypes.
- Two quantitative trait loci (QTL) significantly associated with stem rust resistance were detected using a graph‐based reference.

### 2.3 Genome sequencing

ONT sequencing for Rabiosa was conducted following the method described in Frei et al. (2021). Hi‐C library preparation and sequencing for Rabiosa were performed at the Functional Genomic Center Zurich (FGCZ), Switzerland. Illumina WGS data of Rabiosa were generated by NRGene as described previously (Copetti et al., 2021). For the bi‐parental population, genotyping‐by‐sequencing (GBS) libraries were prepared with the restriction enzyme *Pst*I as described by Begheyn et al. (2018) and sequenced with Illumina HiSeq 2500 at FGCZ.

### 2.4 Rabiosa genome assembly

For Rabiosa v1, ONT long reads were first assembled using Flye 2.8.3 (Kolmogorov et al., 2019) with –min‐overlap 10,000 and –iterations 2, then the contigs were polished with accurate short reads using Polca (Zimin & Salzberg, 2020) for one round. After polishing, haplotigs were removed from the polished assembly using a customized haplotig purge pipeline (Section 2.7). Hi‐C data were mapped to the haplotig‐purged assembly using the Arima Hi‐C mapping pipeline (https://github.com/ArimaGenomics/mapping_pipeline), and SALSA2 (Ghurye et al., 2019) was used to scaffold the haplotig‐purged assembly with the Hi‐C mapping results. The scaffolds generated by SALSA2 were further anchored to pseudo‐chromosomes using ALLMAPS (Tang et al., 2015) based on the genetic linkage map generated with the GBS data from the bi‐parental F_1_ population (Section 2.6). The resulting pseudo‐chromosomes from ALLMAPS were used to anchor the scaffolds generated by SALSA2 again using the Tritex pipeline (Monat et al., 2019) with Hi‐C data, producing the Rabiosa v1 assembly.

For Rabiosa v2, ONT long reads were first corrected with accurate short reads using FMLRC2 (Mak et al., 2023). Then, the corrected ONT long reads were assembled using Canu 2.2 (Koren et al., 2017) with batOptions = ‐eg 0.12 ‐sb 0.01 ‐dg 3 ‐db 3 ‐dr 1 ‐ca 500 ‐cp 50. One round of short‐read polishing was applied to the Canu assembly using Polca (Zimin & Salzberg, 2020). After polishing, the same customized haplotig purge pipeline was applied, and then the haplotig‐purged contigs were scaffolded to pseudo‐chromosomes using the Tritex pipeline with Hi‐C data and Rabiosa v1 as the reference. For the resulting chromosome‐level assembly, a Hi‐C contact map was generated using Juicer (Durand, Shamim, et al., 2016) and 3d‐DNA (Dudchenko et al., 2017), and manual curation was conducted using Juicebox (Durand, Robinson, et al., 2016). This resulted in the Rabiosa v2 assembly.

### 2.5 GBS data pre‐processing

GBS data from the bi‐parental F_1_ population were first demultiplexed using Sabre (Najoshi, 2022), and quality check was performed using FastQC (Andrews, 2015). Later, adapters were trimmed off using Trimmomatic (Bolger et al., 2014).

### 2.6 Genetic linkage map construction

Several genetic linkage maps were constructed in this work. The first genetic linkage map was the one used for scaffolding Rabiosa v1. Clean GBS reads obtained from the above methods were mapped to the scaffolds generated with SALSA2 using the short‐read aligner BWA‐MEM (Li, 2013). Then, single nucleotide polymorphisms (SNPs) were called using SAMtools and BCFtools (Danecek et al., 2021). Ungenotyped SNPs were imputed using LinkImputeR (Money et al., 2017), and the genetic linkage map was built using Lep‐MAP3 (Rastas, 2017) by sorting and ordering markers based on the recombination fraction between SNP markers. When building the genetic linkage map with Lep‐MAP3, Rabiosa was considered as parent 1 and Sikem was considered as parent 2 for all F_1_ individuals, as these were derived from a bi‐directional pair‐wise cross. Informative SNP markers (markers that are heterozygous in either or both parents) were selected for map construction. These SNP markers were separated into seven linkage groups (LGs) using SeparateChromosomes2 from Lep‐MAP3, with lodLimit = 20, *θ* = 0.2.

A second batch of genetic linkage maps was generated based on SNPs called against pseudo‐chromosomes of Rabisoa v2 and the haplotype assemblies (Rabiosa h1 and h2, Section 2.8), following the same read mapping and variant calling methods. Prior to linkage map construction, no imputation was made to fill missing genotypes in the variant calling file, and F_1_ individuals were filtered if more than 30% of their SNPs were not genotyped. Lep‐MAP3 (Rastas, 2017) was again used for linkage map construction. Informative markers from both parents were used for constructing consensus maps, aiming to include as many SNPs as possible into the maps for follow‐up validation. The genetic map distance of the SNP markers was calculated with the given order (the physical position) of the SNP markers from the assemblies (OrderMarkers2 with improveOrder = 0). By comparing the physical position with the genetic map position of the SNP markers, these maps were used to validate the structural correctness of the pseudo‐chromosomes in the assemblies (Figure 4a, track B). If the structure of the pseudo‐chromosome is correct, then the physical position of the markers should correlate well with the corresponding genetic map position. Discrepancies between the physical position and the genetic map position, such as a steep slope in the correlation plot (a short distance on the *x*‐axis, the physical position, corresponds to a much longer distance on the *y*‐axis, the genetic linkage map position, between two markers), might indicate structural mistakes in the assembly.

For the QTL analysis with the graph‐based reference (Section 2.15), SNPs were first identified from the graph‐based reference, and then these SNPs were projected to Rabiosa v2 to obtain their physical positions. These SNPs were genotyped with GBS reads from all F_1_ individuals (Section 2.14), and SNP markers that were only heterozygous in Rabiosa were considered for computing the genetic map position (OrderMarkers2 with improveOrder = 0) based on the projected physical position in Rabiosa v2 using Lep‐MAP3 (Rastas, 2017). To verify the resulting genetic linkage map, a recombination fraction plot was generated using R/qtl (Broman et al., 2003). For the QTL analysis with the single reference (Rabiosa v2), the SNPs obtained when generating the second batch of genetic linkage maps were used for map construction. With Lep‐MAP3 (Rastas, 2017), two genetic linkage maps were constructed with SNP markers that were only heterozygous in Rabiosa or Sikem, respectively. Similarly, the position of SNP markers in the genetic linkage maps was calculated with the given order of these SNP markers in Rabiosa v2.

### 2.7 Customized haplotig purge pipeline

A customized pipeline named PurgeGrass (available at GitHub, https://github.com/Yutang‐ETH/PhaseGrass/tree/main/PurgeGrass) was developed to purge both Flye and Canu assemblies in this work. The pipeline pairs allelic contigs based on evidence from all‐by‐all alignment, micro‐synteny, and Benchmarking Universal Single‐Copy Orthologs (BUSCO) genes. All‐by‐all alignment was conducted using Purge Haplotigs (Roach et al., 2018), and micro‐synteny between contigs was detected using a pipeline consisting of GMAP (Wu & Watanabe, 2005), GffRead (G. Pertea & Pertea, 2020), DIAMOND (Buchfink et al., 2015), and MCScanX (Wang et al., 2012) with transcripts generated in a previous study (Copetti et al., 2021). BUSCO genes were detected using BUSCO v4.1.4 (Manni et al., 2021) with database embryophyta_odb10. A contig is considered as a haplotig of a longer contig if it fulfills one of the following criteria: (1) having alignment coverage greater than 70% with a longer contig, (2) sharing at least five collinear genes with a longer contig, and (3) sharing at least one single‐copy complete BUSCO gene with a longer contig.

### 2.8 Reference‐based phasing and haplotype‐resolved genome assembly

First, WGS Illumina short reads were mapped to Rabiosa v2 pseudo‐chromosomes using BWA‐MEM (Li, 2013), and then SNPs were called using SAMtools and BCFtools (Danecek et al., 2021). Next, Hi‐C data were mapped to Rabiosa v2 pseudo‐chromosomes to phase the SNPs using Hapcut2 (Edge et al., 2016), following the pipeline described here, https://github.com/vibansal/HapCUT2/tree/master/HiC. This resulted in a sparse chromosome‐level phase block per pseudo‐chromosome. In parallel, ONT long reads were mapped to the pseudo‐chromosomes using Winnowmap2 (Jain et al., 2022). By combining the local phase information provided by the ONT read alignment with the long‐range phase information provided by the sparse chromosome‐level phase block derived from Hi‐C data, SNPs were phased through pseudo‐chromosomes using WhatsHap (Martin et al., 2016). This resulted in a dense chromosome‐level phase block per pseudo‐chromosome. Finally, ONT reads were binned to different haplotypes using WhatsHap based on the chromosome‐level phase blocks with high‐density SNPs, resulting in four sets of reads including haplotype 1, haplotype 2, untagged, and unaligned. Only haplotype 1 and 2 reads were used subsequently to generate haplotype assemblies.

Flye (Kolmogorov et al., 2019) was used to assemble reads of each haplotype separately. Then, one round of short‐read polishing was applied to each haplotype assembly using Polca (Zimin & Salzberg, 2020). Next, each assembly was scaffolded with Hi‐C using SALSA2 (Ghurye et al., 2019), and then mis‐joins in the resulting scaffolds were corrected using Tritex (Monat et al., 2019). The corrected scaffolds were aligned to Rabiosa v2 to construct pseudo‐chromosomes using RagTag (Alonge et al., 2022). Additional manual curation to both haplotype assemblies was conducted using Juicer (Durand, Robinson, et al., 2016), 3D‐DNA (Dudchenko et al., 2017), and Juicebox (Durand, Robinson, et al., 2016), resulting in Rabiosa h1 and h2. The two haplotype assemblies were concatenated to a diploid assembly (Rabiosa dip), and a diploid Hi‐C contact map was also generated with Juicer.

### 2.9 Assembly quality assessment

The general assembly statistics were calculated with assembly‐stats (https://github.com/sanger‐pathogens/assembly‐stats). The completeness of the assemblies was assessed using BUSCO v4.1.4 (Manni et al., 2021) with database embryophyta_odb10. The k‐mer profile of each assembly was generated using the k‐mer analysis toolkit (KAT; Mapleson et al., 2017). To calculate the ONT read alignment depth, all ONT long reads were aligned to Rabiosa h1, h2, and dip separately using Winowmap2 (Jain et al., 2022), and the alignment depth was calculated using Mosdepth (Pedersen & Quinlan, 2018). RNA‐seq‐read mapping was done with the methods described in Section 2.10, and the mapping rate was reported from HISAT2 (Kim et al., 2015).

### 2.10 Evidence‐based genome annotation

First, a custom repeat library was generated using RepeatModeler2 (Flynn et al., 2020) and ProtExcluder.pl (Campbell et al., 2014) based on Rabiosa v2. Then, transposable elements (TEs) in Rabiosa v2, h1, and h2 were annotated using RepeatMasker (Smit et al., 2013) with the custom repeat library.

Gene models were built using EVidenceModeler (EVM) (Haas et al., 2008) with evidence from ab initio gene prediction, protein alignment, and transcript alignment following the method described in Tritex (Monat et al., 2019). Ab initio gene prediction was performed using BRAKER2 (Brůna et al., 2021) with protein sequences from Kyuss (Frei et al., 2021). Protein sequences collected from closely related species (Methods S1) were mapped to the assembly using GenomeThreader (Gremme et al., 2005), and alignment results were merged using GenomeTools (Gremme et al., 2013). Transcripts collected from closely related species (Methods S2) were mapped to the assembly using GMAP (Wu & Watanabe, 2005), and mapped transcripts were merged using Cuffcompare (Trapnell et al., 2012) and StringTie merge (M. Pertea et al., 2015). Coding sequences were identified using TransDecoder (Haas, BJ, 2018) with the merged mapped transcripts. Additionally, RNA‐seq data of Rabiosa generated previously (Copetti et al., 2021) were also used for genome annotation in this work. RNA‐seq reads were first trimmed using fastp (S. Chen et al., 2018), then transcripts were constructed using the alignment‐based approach with HISAT2 (Kim et al., 2015) and StringTie2 (M. Pertea et al., 2015) following the instructions detailed in M. Pertea et al. (2016). All evidence was integrated by EVM to generate consensus gene models.

Using BLAST (Altschul et al., 1990), gene models were searched for against The TRansposable Elements Platform (TREP), a TEs database (Schlagenhauf & Wicker, 2016), the UniProt reviewed database (The UniProt Consortium et al., 2021), and the Pfam protein families database (Mistry et al., 2021). Based on the BLAST results, gene models were classified into high‐ and low‐confidence groups. High‐confidence genes were defined as those with a hit in either the UniProt or Pfam database and no more than 25% overlap with a TE gene in TREP. In contrast, low‐confidence genes were defined as those without a BLAST hit in either the UniProt or Pfam database and no more than 25% overlap with a TE gene in TREP. Notably, since the TREP database may not represent all the repetitive sequences of Italian ryegrass, there might be some TE coding genes remaining in the high‐ and low‐confidence groups. A more comprehensive TE database could be used to improve the classification of the functional protein‐coding genes, or TE could be masked prior to gene annotation.

Functional annotation was performed using AHRD (https://github.com/groupschoof/AHRD) and InterProScan 5 (Jones et al., 2014).

### 2.11 Gene‐based synteny analysis

Using Last (Kiełbasa et al., 2011), the high‐confidence genes of Rabiosa v2, h1, and h2 were aligned against genes from the barley cultivar ‘Morex’ (Mascher et al., 2021), Kyuss, a doubled haploid perennial ryegrass genotype (Frei et al., 2021), and Lp261, a self‐compatible perennial ryegrass genotype (Nagy et al., 2022). After alignment, gene‐based synteny was constructed using MCscan (Python version https://doi.org/10.5281/zenodo.594205). The same analysis was applied to check the synteny between Rabiosa v2 and the two haplotype assemblies, and the synteny between Rabiosa h1 and h2. The mean alignment identity of the gene coding sequence between Rabiosa h1 and h2 was calculated with awk commands (see Data Availability Statement) based on the gene coding sequence alignment file generated by Last when running the MCscan pipeline.

### 2.12 Sequence identity between two haplotypes

Pseudo‐chromosomes of Rabiosa h2 (query) were aligned against pseudo‐chromosomes of Rabiosa h1 (reference) using AnchorWave (Song et al., 2022) allowing inversions. The resulting alignments were converted to the Sequence Alignment/Map (SAM) format with the maf‐convert script provided by AnchorWave. Then, the SAM alignment file was further converted to the Pairwise mApping Format (PAF) by paftools.js from Minimap2 (Li, 2018). Next, with the PAF file as input, SyRi (Goel et al., 2019) was used to call SNPs, small insertions and deletions (INDELs), large presence and absence variations (PAVs), and inversions.

In parallel, short reads were aligned to Rabiosa h1 to call SNPs and small INDELs with BWA (Li, 2013) and BCFtools (Danecek et al., 2021). ONT long reads were aligned to Rabiosa h1 with Winnomap2 (Jain et al., 2022), and PAVs were called with Sniffles (Sedlazeck et al., 2018). Gaps in Rabiosa h1 and h2 were detected using a custom Python script. Common SNPs, small INDELs, and large PAVs that were detected by both whole‐genome alignment (WGA) and read alignment were identified by custom R scripts. Common SNPs and INDELs between two variant calling methods were defined as SNPs and INDELs sharing the exact start position, while common PAVs between two variant calling methods were defined as PAVs that overlap each other, or the start position of one PAV is within 10 bp downstream of the end position of the other PAV. Only common variants were used for sequence identity calculation and PAVs containing gaps were filtered. Sequence identity was calculated per 100 kb along the pseudo‐chromosome as (100 kb − total length of common variants − gaps − unaligned sequences reported by SyRi)/100 kb.

### 2.13 Constructing graph‐based reference

Pair‐wise WGA was first performed among Rabiosa v2, h1, and h2 using AnchorWave (Song et al., 2022). Then, the resulting alignments were converted to PAF format as described above, and one PAF file was obtained for each pair‐wise alignment. All PAF files were concatenated into one PAF file using a custom R script and then fed into pangenome graph builder (PGGB, Garrison et al., 2023) to construct the graph‐based reference.

### 2.14 Genotyping of SNPs in the graph‐based reference

SNPs in the graph‐based reference were detected by deconstructing the graph‐based reference using vg deconstruct (Garrison et al., 2018). Subsequently, the resulting vcf file from deconstruction was filtered using vcfbub (https://github.com/pangenome/vcfbub), resulting in a vcf file containing only SNPs. GBS data of each F_1_ individual in the bi‐parental population were mapped to the graph‐based reference using Giraffe (Sirén et al., 2021), and the resulting alignment files were further processed with the vg toolkit (Garrison et al., 2018). Based on the alignment, any SNPs in the previous vcf file covered by at least five GBS reads of one F_1_ individual were genotyped by the vg toolkit, resulting in one vcf file with the genotypes of this individual. All individual vcf files were finally merged to one vcf file using BCFtools merge (Danecek et al., 2021).

### 2.15 Phenotyping and QTL analysis of stem rust resistance

The F_1_ population was grown at the same location (47.427° N, 8.516° E, Zurich, Switzerland) but in a different neighboring field (environment) for each of the three phenotyping years (2019, 2020, and 2021). This design was intended to maximize the likelihood of exposure to the same stem rust strain across years, although the composition of the field isolates was not determined. In spring 2019, the F_1_ plants were cut after the end of heading. In the second growth, the seed harvesting date was determined based on the cumulative average daily temperature measured 5 cm above the ground after the start of flowering for each plant individually. Once a plant reached the temperature sum of around 590°C, the plant was harvested by cutting with a sickle and phenotyped for stem rust occurrence. Stem rust occurrence was scored on each plant using a scale from 1 to 9, where 1 = no rust occurrence, 2 = trace of rust, 3 = 5%, 4 = 10%, 5 = 25%, 6 = 40%, 7 = 60%, 8 = 75%, and 9 = more than 75% of the plant is covered with rust (Schubiger & Boller, 2016). To analyze the phenotypic data across environments, the following linear mixed model was used:

(1) $$y_{ijkl}=\mu +g_{i}+e_{j}+g_{eij}+b_{lkj}+\varepsilon_{ijkl}$$

where *y_ijkl_* represents the measurement for stem rust occurrence on a single plant basis, *μ* denotes the overall mean, *g_i_* the effect of genotype *i*, *e_j_* the effect of environment *j*, *ge_ij_* the interaction effect of genotype *i* with environment *j*, *b_lkj_* the effect of the *l*th incomplete block nested within the *k*th complete block, and *ε_ijkl_* the residual error. The best linear unbiased estimators (BLUEs) for each genotype across all environments were calculated by using all factors within Equation (1) as random effect, except *b_lkj_*, which was considered as fixed effect. All the phenotypic data analyses were conducted using R version 4.3.1 (R Core Team, 2023). Package lm4 (Bates et al., 2015) was used for fitting mixed‐effect models. Package emmeans (Searle et al., 1980) was used for calculating the BLUEs. Resistance data were obtained for 133 F_1_ individuals along with their two parents, and the BLUEs calculated for the 133 F1 individuals were used for QTL analysis.

The genetic linkage maps (Section 2.6) and the BLUEs were integrated into a CSV file as input for QTL analysis using R/qtl (Broman et al., 2003). Composite interval mapping (CIM) was used to calculate the marker‐trait association (function cim in R/qtl with n.marcovar = 5, window = 10). The suggested logarithm of odds (LOD) threshold was defined as the 95th percentile of the distribution of the genome‐wide maximum LOD score. The distribution was obtained by conducting a permutation test using the function scanone from R/qtl with n.perm = 200. The percentage of variance explained by each QTL was calculated using the function makeqtl from R/qtl.

## 3 RESULTS

### 3.1 Whole‐genome sequencing and reduced representation sequencing of Italian ryegrass

For Rabiosa, the following sequencing data were obtained: ONT long reads (total length of 153 Gb, corresponding to ∼60‐fold coverage of the haploid genome; read N50 of 21 kb), Hi‐C reads (540 million 150 bp pair‐end reads, corresponding to ∼60‐fold coverage of the haploid genome), and Illumina short reads (818 million 150 bp pair‐end reads, corresponding to ∼85‐fold coverage of the haploid genome) from a previous study (Copetti et al., 2021).

The F_1_ population, together with the two parental genotypes Rabiosa and Sikem, was subjected to GBS, yielding on average 2.6 million 90‐bp single‐end reads per individual (ranging from 0.1 to 6 million) across the 304 F1 individuals.

### 3.2 Chromosome‐level unphased haploid assembly of Rabiosa

For the chromosome‐level haploid assembly (Rabiosa v1), the raw Flye assembly was first polished with accurate short reads, resulting in contigs with a total length of 3.39 Gb (Table S1) and a contig N50 of 660.61 kb (Table S1). Next, haplotigs in the polished assembly were removed with a customized pipeline, resulting in a haplotig‐purged assembly with its total length reduced to 2.75 Gb (Table 1). Based on the haplotig‐purged assembly, subsequent scaffolding with a consensus genetic linkage map containing 26,203 informative SNP markers (2083 and 2293 markers with a unique genetic map position in the map of parent 1 and 2, respectively, Figure S1) and Hi‐C data generated a chromosome‐level assembly with a scaffold N50 of 275.65 Mb and a 77.82% anchoring rate (2.14 out of 2.75 Gb were anchored to seven pseudo‐chromosomes, Table 1).

**TABLE 1:** Assembly statistics of Rabiosa assemblies.

	Rabiosa v1	Rabiosa v2	Rabiosa h1	Rabiosa h2
Total assembly size (Gb)	2.75	2.46	1.78	1.74
Contig N50 (kb)/L50 (#)	660.61/1350	1332.60/563	276.06/1807	265.82/1808
Contig N90 (kb)/L90 (#)	125.25/5908	396.90/1838	77.53/6528	74.79/6573
Scaffold N50 (Mb)/L50 (#)	275.65/5	338.75/4	247.62/4	245.51/4
Scaffold N90 (Mb)/L90 (#)	0.18/786	244.92/7	186.44/7	178.87/7
Total length anchored to pseudo‐chromosomes (Gb)	2.14	2.24	1.67	1.63
Anchoring rate (%)	77.79	91.16	94.04	93.85
Total BUSCO (%)	94.00	94.60	89.70	89.00
Single‐copy BUSCO (%)	88.20	89.00	83.60	83.60
Duplicated BUSCO (%)	5.80	5.60	6.10	5.40
Fragmented (%)	0.80	0.40	0.30	0.60
Missing (%)	5.20	5.00	10.00	10.40

Abbreviation: BUSCO, Benchmarking Universal Single‐Copy Orthologs.

The more contiguous chromosome‐level haploid assembly (Rabiosa v2) was generated by scaffolding based on Rabiosa v1 with contigs produced by Canu (Koren et al., 2017), using the same ONT data. Contigs generated by Canu were first polished, resulting in a total length of 4.18 Gb (Table S1) and a higher contig N50 of 966.70 kb (Table S1) compared to Rabiosa v1. Removing redundant haplotigs resulted in a haplotig‐purged assembly with a total length of 2.46 Gb (Table 1) and a higher contig N50 of 1.33 Mb (Table 1). Using Rabiosa v1 as the reference together with Hi‐C data, this haplotig‐purged assembly was scaffolded, resulting in a chromosome‐level assembly with a scaffold N50 of 338.75 Mb and a 91.16% anchoring rate (2.24 out of 2.46 Gb was anchored to seven pseudo‐chromosomes, Table 1). Rabiosa v2 was used for all further analyses.

### 3.3 Chromosome‐level haplotype‐resolved assembly of Rabiosa

**FIGURE 1:**
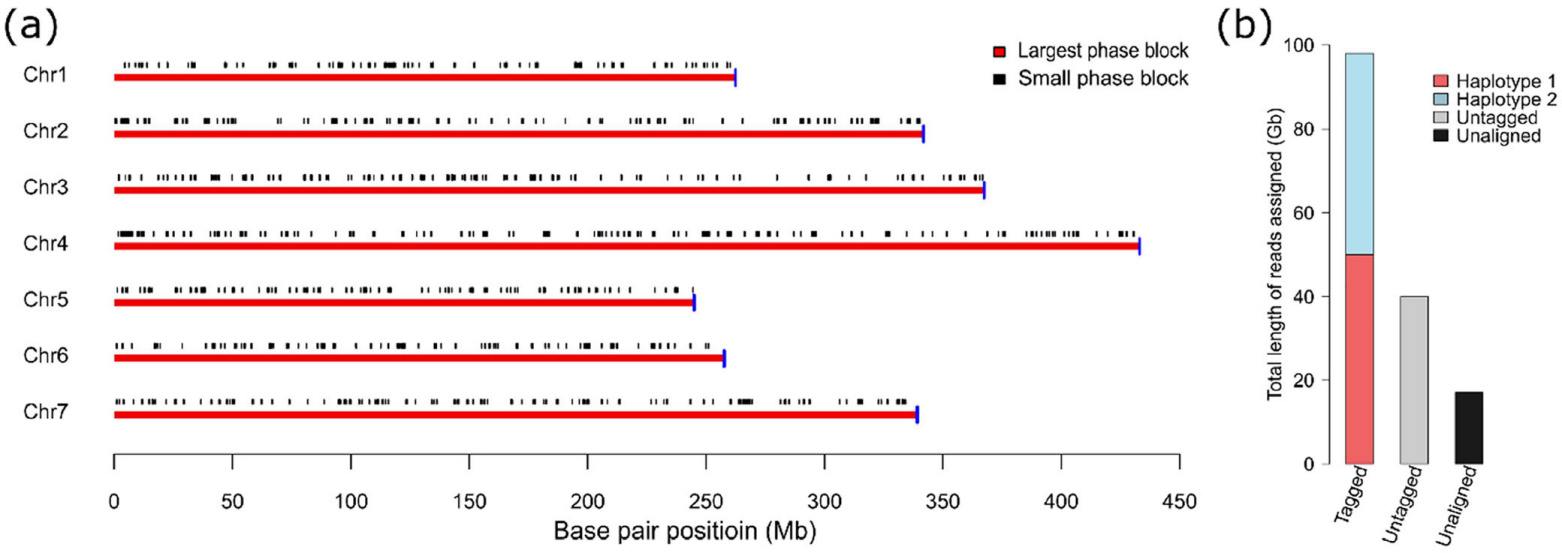
(a) Phase blocks generated by reference‐based phasing. The *y*‐axis indicates the seven pseudo‐chromosomes (Chr 1–7), and the *x*‐axis indicates the base pair position (in Mb) in the pseudo‐chromosome. In each pseudo‐chromosome, the red line represents the largest phase block, and black vertical lines indicate small phase blocks that could not be integrated into the largest phase block. The blue vertical line indicates the length of the pseudo‐chromosome, showing whether the largest phase block spans the whole pseudo‐chromosome and reaches chromosome‐level coverage. (b) Read binning results from reference‐based phasing. Reads assigned to haplotypes were classified as tagged, reads mapped to pseudo‐chromosomes of Rabiosa v2 but not assigned to either haplotype were classified as untagged, and reads not mapped to any pseudo‐chromosomes were classified as unaligned. The sum of tagged, untagged, and unaligned reads is equal to the total length of the Oxford Nanopore Technologies (ONT) reads used for reference‐based phasing.

With the unphased haploid assembly Rabiosa v2, a reference‐based phasing approach was applied to separate the reads belonging to the two Rabiosa haplotypes. Seven chromosome‐level phase blocks together with some small phase blocks that could not be integrated into the chromosome‐level phase blocks were constructed with high‐density SNPs (Figure 1a; Table S2). Ideally, one chromosome‐level phase block should be obtained for each pseudo‐chromosome, and the small phase blocks might lead to haplotype switches in downstream haplotype partitioning (assigning long reads to corresponding haplotypes). Based on the haplotypes of all the phase blocks, ONT reads were assigned to one of the four groups: haplotype 1, haplotype 2, untagged, and unaligned (Figure 1b, Table S3). Roughly 50 Gb ONT long reads (∼20‐fold coverage) were assigned to either haplotype, and based on these haplotype‐specific read sets, two phased haploid assemblies were generated. Each haplotype assembly showed a total size of ∼1.80 Gb and a contig N50 of ∼270.00 kb (Table 1). By further scaffolding, around 1.60 Gb of each assembly was anchored to seven pseudo‐chromosomes, resulting in two chromosome‐level assemblies with a scaffold N50 of around 250.00 Mb (Table 1). The two chromosome‐level phased assemblies were named Rabiosa h1 and Rabiosa h2, respectively.

### 3.4 Quality check of the Rabiosa assemblies

**FIGURE 2:**
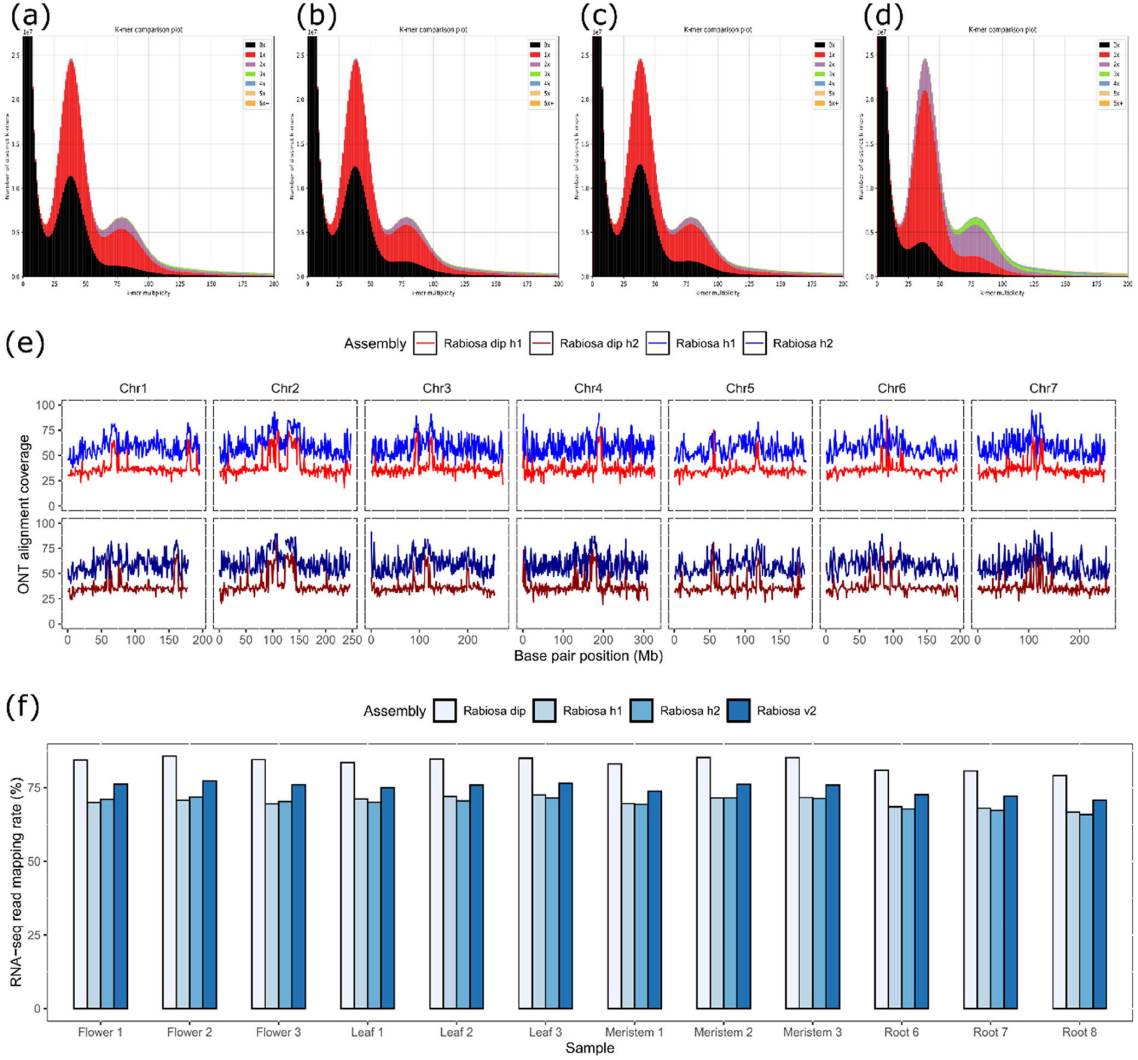
(a–d) K‐mer comparison between assembly and whole‐genome sequencing (WGS) short reads for Rabiosa v2, Rabiosa h1, Rabiosa h2, and Rabiosa dip, respectively. The plots show whether the presence and copy number of the k‐mers in the assembly match the distribution of the k‐mers in WGS short reads. The *x*‐axis represents k‐mer frequency in the WGS short reads, and the *y*‐axis shows the corresponding count of the distinct k‐mers from the reads. For a heterozygous diploid genome, two peaks are expected for the distribution of the k‐mers from reads. The first peak (heterozygous peak) at the lower frequency represents k‐mers from the two alleles at heterozygous loci, and the second peak (homozygous peak) at the doubled frequency of the first peak represents k‐mers from homozygous loci. The colors in the plot show the copy number of k‐mers from the reads in the assembly, with black showing no presence, red showing one copy, and purple showing two copies. For a haploid assembly with only one allele present for each locus, the first peak should be half black and half red, and the second should be full red. For a diploid assembly with both alleles present for each locus, the first peak should be full red, and the second peak should be full purple. (a–c) suggest that Rabiosa v2, h1, and h2 are haploid assemblies with some homozygous sequences missing (small black area under the homozygous peak). (d) suggests that Rabiosa dip is a diploid assembly with some alleles from heterozygous regions missing (small black area under the heterozygous peak) and some homozygous regions collapsed as one copy (red area under the homozygous peak). (e), Oxford Nanopore Technologies (ONT) read alignment coverage for Rabiosa h1 (blue), haplotype 1 in Rabiosa dip (red), Rabiosa h2 (dark blue), and haplotype 2 in Rabiosa dip (dark red). The read alignment coverage for a haplotype in the diploid assembly is expected to be half of the alignment coverage of a haploid assembly. (f) RNA‐seq read mapping rate (*y*‐axis) in Rabiosa v2, h1, h2, and dip. RNA‐seq data were obtained from multiple tissues with three replicates (*x*‐axis).

Quality assessments were conducted for the unphased haploid assembly (Rabiosa v2) and the two phased assemblies (Rabiosa h1 and h2). All three assemblies showed a high completeness as indicated by their total BUSCO scores (Table 1) and the k‐mer comparison results (Figure 2a–c). All three assemblies reached a chromosome‐level contiguity as indicated by their scaffold N50 (Table 1). Notably, Rabiosa v2 showed a higher completeness and contiguity compared to the two‐phased assemblies, reflected by its larger assembly size and higher scaffold N50 (Table 1).

Rabiosa h1 and h2 were concatenated to form a diploid assembly (Rabiosa dip). Rabiosa dip showed a typical diploid assembly k‐mer profile (Figure 2d), suggesting that it was a good representation of the diploid genome. For each haplotype in the diploid assembly, a halved ONT read alignment coverage was observed compared to the haploid assemblies (Figure 2e), suggesting that the haplotypes of Rabiosa were indeed separated. Compared to the three haploid assemblies, Rabiosa dip showed a higher total BUSCO score and, as expected, a much higher duplicated BUSCO score (Table S4) as well as a higher RNA‐seq read mapping rate (Figure 2f). This suggested that the haplotype‐resolved diploid assembly was a more complete representation of the diploid genome when compared to the haploid assemblies.

**FIGURE 3:**
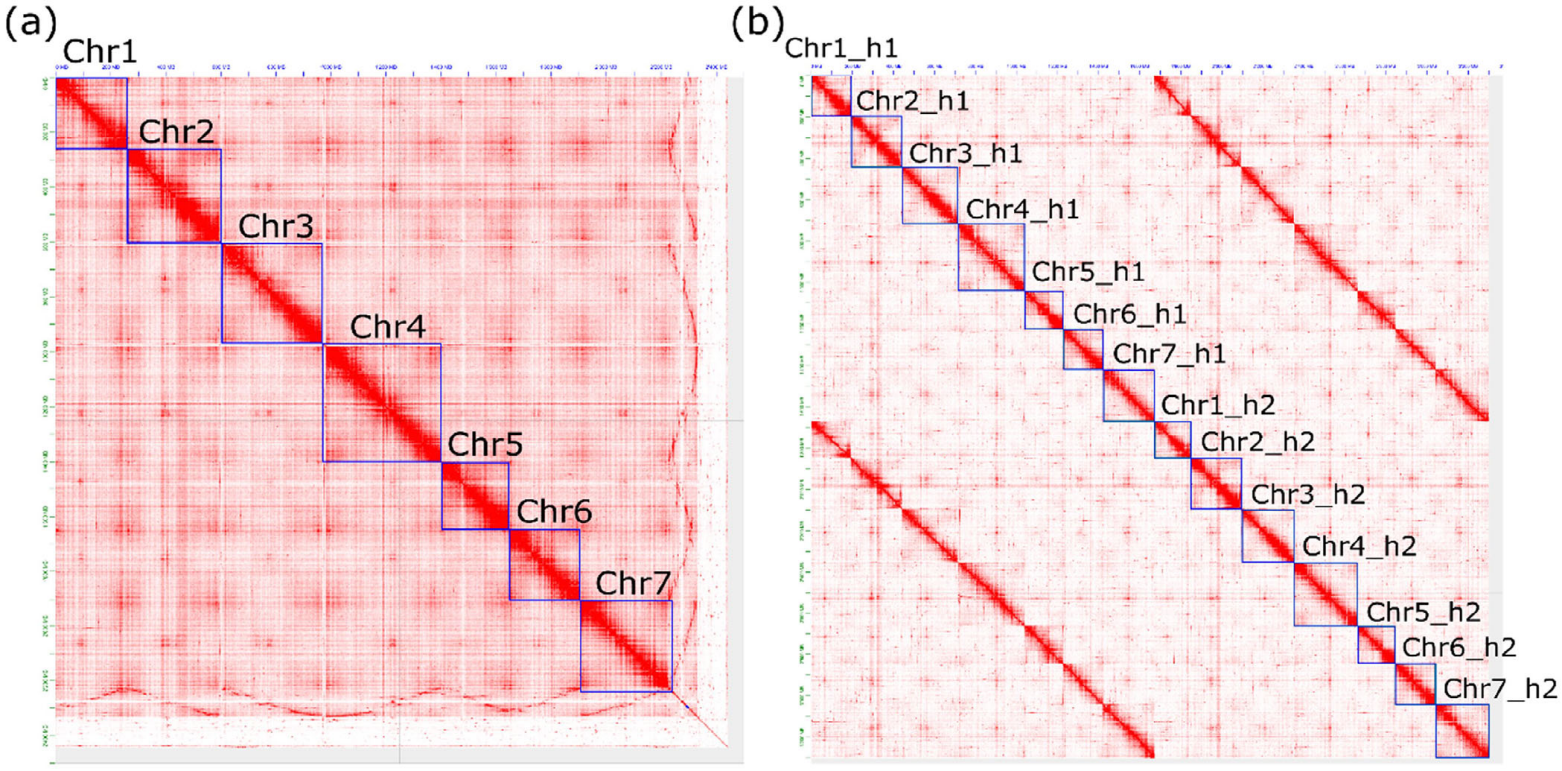
(a) High‐throughput chromosome conformation capture (Hi‐C) contact map of Rabiosa v2. (b) Hi‐C contact map of pseudo‐chromosomes of both haplotypes in Rabiosa dip. Each red pixel in the plot indicates one interaction between two loci within or between chromosomes, and each blue box represents one pseudo‐chromosome. If the sequence in the pseudo‐chromosome is correctly ordered and oriented, then more interactions should be observed between closely linked loci within chromosomes, resulting in a “smooth” red diagonal line.

**FIGURE 4:**
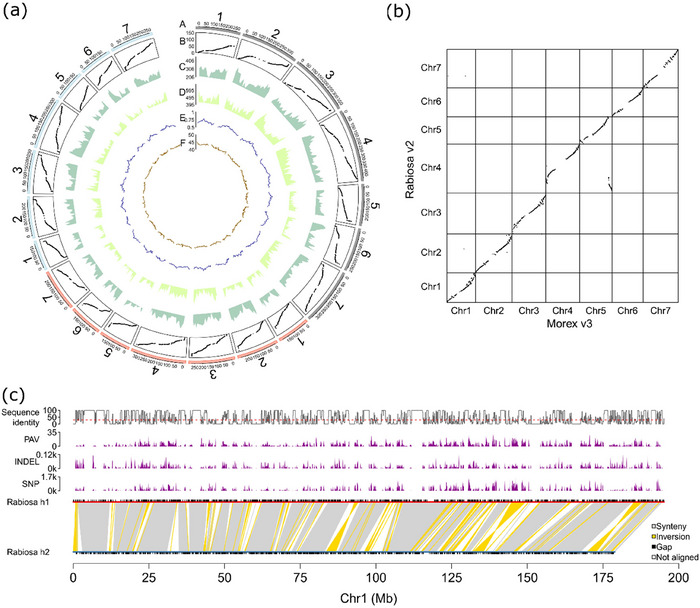
(a) Circos plot showing genomic features of Rabiosa v2, h1, and h2. Track A shows pseudo‐chromosomes of Rabiosa v2 (gray), h1 (red), and h2 (blue), respectively; track B shows the base pair position in the assembly versus genetic linkage map position; track C shows the number of high‐confidence genes per 10 Mb; track D shows the number of low‐confidence genes per 10 Mb; track E shows the fraction of repetitive sequences per 10 Mb; and track F shows the GC content per 10 Mb. (b) Genome synteny between Rabiosa and barley cultivar Morex. (c) Sequence variation between Rabiosa h1 and Rabiosa h2. The distribution of gaps, single nucleotide polymorphisms (SNPs), insertions and deletions (INDELs), and presence and absence variations (PAVs) are displayed. Sequence identity per 100 kb was revealed by the track at the top with the red dashed line indicating 30% identity.

The order and orientation of the contigs in the pseudo‐chromosomes of Rabiosa v2, h1, and h2 were found to be correct based on the Hi‐C contact maps (Figure 3a,b). Complementary to the Hi‐C contact maps, large‐scale structural correctness of the pseudo‐chromosomes was also supported by the high consistency between marker order in the genetic map and their physical position in the assembly (Figure 4a, track B).

### 3.5 Genome annotation and gene‐based synteny analyses

With the evidence‐based genome annotation pipeline, a total of 72,069, 56,142, and 55,928 high‐confidence genes were identified for Rabiosa v2, h1, and h2, respectively (Figure 4a, track C). In addition, 113,004, 91,753, and 90,448 low‐confidence genes were identified for Rabiosa v2, h1, and h2, respectively (Figure 4a, track D). With all the gene models (including high‐ and low‐confidence genes), a high completeness of the gene space was observed in Rabiosa v2, h1, and h2, as indicated by the total complete BUSCO scores of 91.8%, 85.7%, and 85.5%, respectively (Table S4). The number of complete BUSCO genes found in high‐confidence and low‐confidence genes was 83.3% and 9.2% in Rabiosa v2, respectively (Table S4), indicating the presence of true genes in the low‐confidence gene set. Around 70% of each assembly was found to be composed of repetitive sequences (Figure 4a, track E; Table S5), and an average GC (guanine‐cytosin) content of 44% was observed for each assembly (Figure 4a, track F).

Based on the high‐confidence gene models, a highly consistent gene‐based synteny was observed between Rabiosa and closely related species, including perennial ryegrass (*Lolium perenne* L.) and barley (Figure 4b; Figure S2). The known translocation resulting in chromosome 4 of *Lolium* spp. having synteny with chromosome 4 and a segment of chromosome 5 of barley (Pfeifer et al., 2013) was observed (Figure 4b), validating the correctness of the pseudo‐chromosome structure in the Rabiosa assemblies. Notably, the inverted orientation of chromosomes 6 and 7 between Rabiosa and Kyuss (Figure S2) does not indicate true chromosome rearrangements between Italian and perennial ryegrass but is indicative for the wrong orientation of pseudo‐chromosomes in the first version of Kyuss (Frei et al., 2021), as revealed by Y. Chen et al. (2024). A highly consistent chromosome‐level gene order was observed between the Rabiosa haplotypes h1 and h2 (Figure S3), with a mean alignment identity of 98.22% across 13,308 gene pairs.

### 3.6 High sequence variation between the two haplotypes of Rabiosa

With a k‐mer‐based analysis, we first estimated the level of heterozygosity of Rabiosa, and a high level of heterozygosity of 3.43% was observed (Figure S4). This suggests a high sequence variation between the two haplotypes of Rabiosa with three to four SNPs per 100 bp. In parallel to the k‐mer‐based analysis, we also tried to estimate the sequence variation between the two haplotypes of Rabiosa based on not only SNPs, but also other types of variants called from WGA and read mapping. A total of 2,205,246 SNPs, 181,734 small INDELs (length shorter than 50 bp), and 52,578 large PAVs (length equal to or greater than 50 bp) were detected between Rabiosa h1 and h2 (Figure 4c; Table S6). With these variants, sequence identity between haplotypes was calculated per 100 kb window along the pseudo‐chromosomes, and sequence identity as low as 30% was observed between haplotypes in many 100 kb windows (Figure 4c; Figure S5). This low sequence identity suggests that there was a much higher level of sequence variation between the two haplotypes of Rabiosa when structural variants were also considered.

### 3.7 QTL analysis for stem rust resistance with a graph‐based reference of Rabiosa

QTL analysis for stem rust resistance was conducted with the genetic linkage map constructed based on the graph‐based reference and compared to QTL results from a genetic linkage map constructed based on a single reference (Rabiosa v2).

**FIGURE 5:**
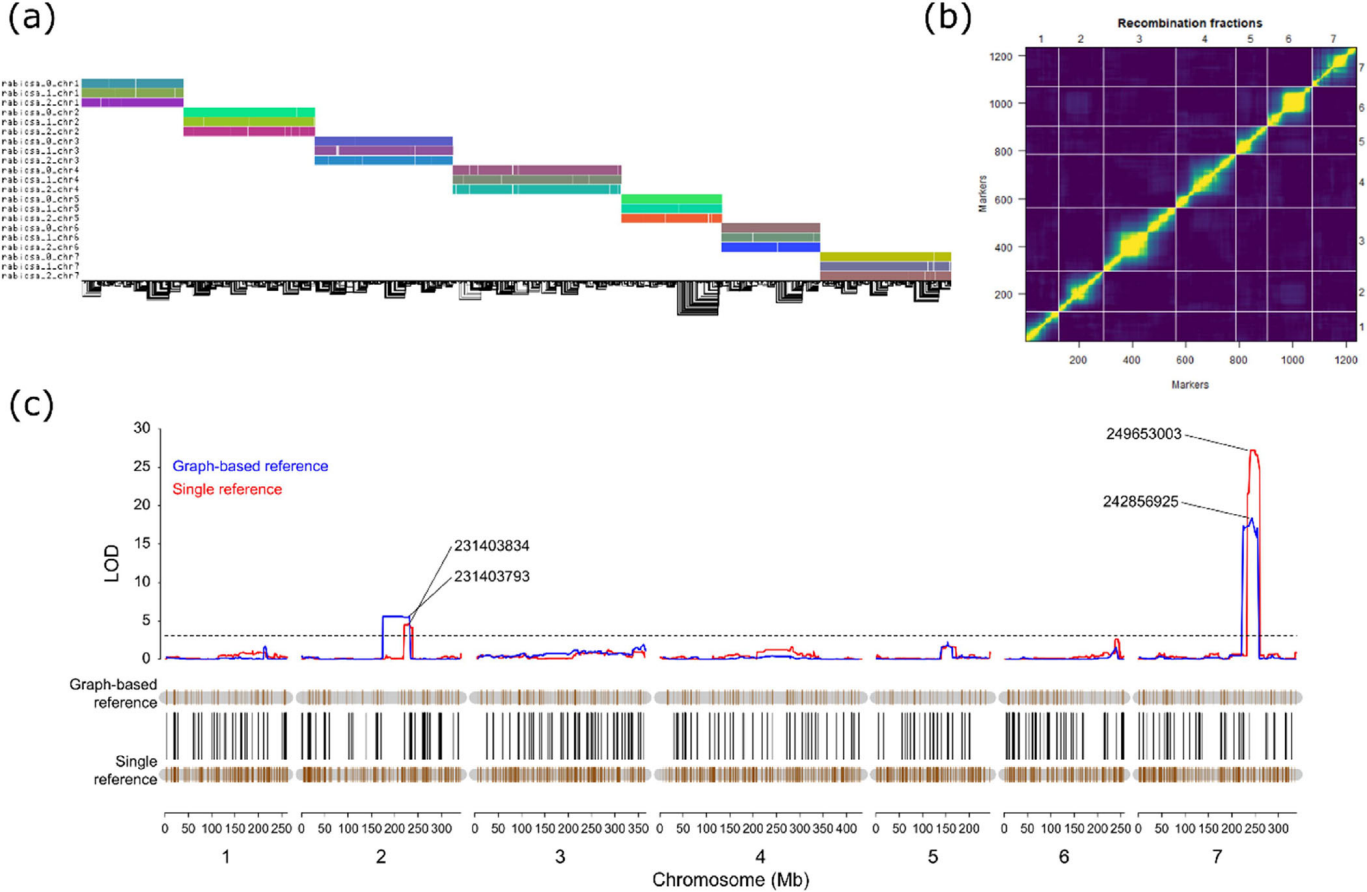
(a) The 1D visualization of the graph‐based reference of Rabiosa showing multiple alignments for Rabiosa v2, h1, and h2. Each horizontal colored rectangle corresponds to a pseudo‐chromosome in one assembly (one path in the graph) containing nodes in the graph. The name of the pseudo‐chromosome was shown by the *y*‐axis label (rabiosa_0, rabiosa_1, and rabiosa_2 correspond to Rabiosa v2, h1, and h2, respectively). The black lines at the bottom are the edges in the graph indicating how nodes were connected. (b) Pair‐wise recombination fraction between markers in the Rabiosa linkage map generated with single nucleotide polymorphisms (SNPs) from the graph‐based reference. A low recombination fraction (indicated by the yellow color) was observed within linkage groups, and a high recombination fraction (no linkage, indicated by the dark blue color) was observed between linkage groups and distant parts of the same linkage group. (c) Results of quantitative trait locus (QTL) analysis for stem rust resistance with a graph‐based reference and a single reference (Rabiosa v2). The brown vertical lines in the gray horizontal bars (chromosomes) represent SNPs used for QTL analysis, and black lines connect the same SNP between the graph‐based reference and the single reference. The numbers linked to the significant QTL peaks show the base pair position (in Rabiosa v2) of the most significant SNP in the QTL.

The graph‐based reference was constructed based on the three Rabiosa assemblies (Rabiosa v2, h1, and h2). Rabiosa v2 was included in the graph‐based reference so that variants detected from the graph could be projected to Rabiosa v2 to find their corresponding physical positions in Rabiosa v2. The physical position from the same coordinate system (the same assembly) allowed us to compare the QTL analysis results between the single‐reference and the graph‐based reference. The graph‐based reference contained 21 paths, corresponding to the 21 pseudo‐chromosomes of the three assemblies. The total size of the graph‐based reference was 3.66 Gb, and there were 243,499,034 nodes and 334,693,667 edges in the graph (Figure 5a).

Since the graph‐based reference is essentially a form of non‐redundant multiple sequence alignment, variants can be directly detected from the graph‐based reference. With the graph‐based reference of Rabiosa, 54,614 SNPs with alleles differentiating haplotypes were obtained and projected to Rabiosa v2 to have the corresponding physical positions in Rabiosa v2. These SNPs were further genotyped with GBS reads from all F_1_ individuals and both parents. Phenotypic data of stem rust resistance were available for 133 F_1_ individuals and the two parents (Rabiosa and Sikem), and a clear segregation of stem rust resistance was observed as a bimodal distribution (Figure S6). After genotyping, 304 F_1_ individuals with 1,237 SNPs that were only heterozygous in Rabiosa were selected to build a genetic linkage map (Figure 5b). Combining the genetic linkage map and the phenotypic data of stem rust resistance resulted in 132 F_1_ individuals for subsequent QTL analysis. Two significant QTL associated with stem rust resistance were detected, with one located on LG 2 and the other located on LG 7, corresponding to pseudo‐chromosomes 2 and 7 in Rabiosa v2, respectively (Figure 5c; Table 2).

**TABLE 2:** Quantitative trait locus (QTL) analysis results for stem rust resistance.

QTL	Reference	Left boundary (bp)	Right boundary (bp)	Highest LOD	LOD threshold	Variance explained (%)
qtl.chr2	Rabiosa v2	220,462,590	237,911,985	4.64	2.71	5.93
qtl.chr7	Rabiosa v2	233,515,340	259,843,203	27.52	2.71	47.18
qtl.chr2	Graph based	174,459,966	231,403,834	5.66	2.71	4.83
qtl.chr7	Graph based	224,224,230	255,175,175	18.39	2.71	35.30

Abbreviation: LOD, logarithm of odds.

For QTL analysis with the single reference, a genetic linkage map was constructed based on Rabiosa v2. GBS reads from all F_1_ individuals and both parents were aligned to Rabiosa v2 to call SNPs. In total, 264 F_1_ individuals with 5,034 SNPs that were only heterozygous in Rabiosa were used to construct the genetic linkage map. Combining this genetic linkage map with the phenotypic data of stem rust resistance resulted in 120 individuals for QTL analysis. The same two QTL that were associated with stem rust resistance were detected (Figure 5c; Table 2), validating the results of the QTL analysis with the graph‐based reference. In addition, a QTL analysis was also conducted using a linkage map consisting of 6,778 SNPs that were only heterozygous in Sikem. In this analysis, both QTL, although detectable, did not exceed the significance threshold (Figure S7). Combining this result with the phenotypic distribution of resistance to stem rust (Figure S6), we hypothesize that complementing resistance sources, either another dominantly inherited resistance allele of the QTL on chromosome 7 or the independently segregating resistance source chromosome 2 from Sikem, are segregating in this population.

## 4 DISCUSSION

Rabiosa v2, the chromosome‐level unphased haploid genome assembly of the highly heterozygous Italian ryegrass genotype Rabiosa, showed a much better quality compared to the reported draft assemblies for this genotype (Copetti et al., 2021) or other genotypes of this species (Knorst et al., 2019). It showed a comparable quality to the recently reported reference‐level assembly (Brunharo et al., 2025) with regard to the scaffold contiguity and assembly completeness (Rabiosa v2 vs. Brunharo's assembly, Scaffold N50, 338.75 Mb vs. 363.00 Mb, total BUSCO score, 94.60% vs. 92.80%). Notably, for Rabiosa v2, 60‐fold error‐prone ONT data were used to generate the assembly, while for the assembly of Brunharo et al. (2025), nearly 100‐fold accurate PacBio HiFi data were used. More importantly, we generated two haplotype assemblies (Rabiosa h1 and h2, Figures 1, 2, and 3b) with the error‐prone ONT data, which is more challenging to work with compared to the highly accurate PacBio HiFi data (Li & Durbin, 2024). Although the contigs of each haplotype assembly we generated were rather fragmented (contig N50 270 kb, Table 1), we still managed to anchor more than 93% of the total length of the contigs onto pseudo‐chromosomes (Table 1). Besides, when concatenating the two haplotype assemblies into a diploid assembly (Rabiosa dip) and comparing it with the haploid assemblies, we found that the diploid assembly was a more complete representation of the gene space of the diploid genome (higher total BUSCO score and RNA‐seq read mapping rate, Table S4; Figure 2f). This suggests that a haplotype‐resolved assembly should be considered when generating genome assemblies for highly heterozygous species such as Italian ryegrass.

A high level of heterozygosity was estimated with k‐mers for Rabiosa (3.43%, Figure S4), which is consistent with the high SNP frequency (1 per 20 bp) observed in ryegrass (Hayes et al., 2013). Compared to other published highly heterozygous plant genomes, such as tea (*Camellia sinensis*, heterozygosity 2.31%, Zhang et al., 2021), lychee (*Litchi chinensis*, heterozygosity 2.27%, Hu et al., 2022), and diploid potato (*Solanum tuberosum*, heterozygosity 2.10%, Q. Zhou et al., 2020), Rabiosa showed the highest level of heterozygosity, suggesting that there is a high level of sequence variation between the two haplotypes of Rabiosa. Indeed, a very high level of sequence variation (30% sequence similarity in some 100 kb regions) was observed between the two haplotypes of Rabiosa when estimating the sequence variation with different types of variants, including SNPs, INDELs, and PAVs (Figure 4c). As the mean alignment identity of the gene coding sequence between haplotypes is around 98%, as revealed by the gene‐based synteny analysis, the high sequence variation between the haplotypes is mainly from intergenic regions. This is consistent with the high intergenic sequence variation found previously in Rabiosa based on a single locus analysis (Copetti et al., 2021). Such a high level of sequence variation might explain some of the challenges or technical limitations we encountered when assembling Rabiosa.

We found that the high level of sequence variation between the two haplotypes of Rabiosa may cause sequence‐alignment‐based haplotig‐purge tools, such as Purge Haplotigs (Roach et al., 2018) and purge_dups (Guan et al., 2020), to fail to align allelic contigs, leaving more redundant allelic sequences (false duplication) in the assembly after purging (indicated by the duplicated BUSCO score, Table S4). The same situation was observed in other *Lolium* genome assemblies. For example, a haploid *L. rigidum* assembly with 70% duplicated BUSCO score (Paril et al., 2022) was generated using Purge Haplotigs (Roach et al., 2018), and the new reference‐level Italian ryegrass assembly with 20% duplicated BUSCO score (Brunharo et al., 2025) was generated using purge_dups (Guan et al., 2020). Notably, for the Brunharo's assembly, even though 100‐fold high‐quality PacBio HiFi data were used for assembly, the relatively high unexpected rate of false duplication remained. This suggests that the false duplication in the assembly is not a limitation from the input sequencing data but a technical limitation of the bioinformatic methods that could not handle the high sequence variation between haplotypes. To overcome the technical limitation and purge as many redundant haplotigs as possible for Rabiosa, in addition to sequence‐alignment‐based methods, we also used micro‐synteny and BUSCO genes to pair allelic contigs, which led to better purging results compared to using the sequence‐alignment‐based tools alone (lower duplicated BUSCO scores, Table S4).

Genome phasing was also hampered by the high sequence variation between haplotypes. A reference‐based phasing approach was chosen in this work to separate the haplotypes of Rabiosa as no parental datasets or highly accurate long reads were available. Based on the unphased haploid assembly (Rabiosa v2) as the reference, we found that around 26% (40 Gb out of the total 153 Gb) of the ONT reads were left unassigned to any haplotypes based on the long‐read alignment to the reference. This high proportion of unassigned reads could be attributed to reference bias caused by the high sequence variation between haplotypes rather than sequences from homozygous regions (Porubsky et al., 2021). As the haploid assembly (Rabiosa v2) was a mosaic representation of the diploid genome with alleles alternating between haplotypes in the pseudo‐chromosomes, when mapping long reads from both haplotypes to the reference, reads from one haplotype might be aligned to the reference, but reads from the other haplotype may not be aligned or incorrectly aligned to the reference (Jain et al., 2022). If reads were incorrectly aligned to the wrong position due to repetitive sequences (Jain et al., 2022), they might be left unassigned to any haplotypes because of no variants called in the repetitive sequences (Figure S8). The high proportion of unassigned reads led to a low coverage (∼20‐fold) of haplotype‐tagged reads in each haplotype, which compromised the contiguity and completeness of the phased haplotype assemblies (Rabiosa h1 and h2). For the reference‐based phasing approach, due to the reference bias caused by the high sequence variation between haplotypes, long reads can only be assigned to haplotypes when they are correctly aligned to the reference. More future work is needed to overcome this limitation when applying reference‐based phasing to highly heterozygous genomes. Nonetheless, to the best of our knowledge, the two haplotype assemblies (Rabiosa h1 and h2) generated in this work are the first and the only haplotype‐resolved assembly reported for Italian ryegrass so far.

A graph‐based reference can be built by adding structural variations (SVs) into a single reference, and this type of graph‐based reference has been used for many genome‐wide association studies (GWAS) in plants (J. Chen et al., 2023; Cochetel et al., 2023; Liu et al., 2020; Y. Zhou et al., 2022). A graph‐based reference can also be built by aligning chromosome‐level genome assemblies, and this approach is more accurate than adding a catalog of SVs to a single reference (Hickey et al., 2020). However, so far, not many attempts have been made to perform GWAS or QTL analysis in plants with this type of graph‐based reference built from de novo assemblies (Vaughn et al., 2022). To explore the potential of the graph‐based reference built from de novo assemblies for future pangenomic studies in *Lolium* spp., we generated a graph‐based reference with all three Rabiosa assemblies (Rabiosa v2, h1, and h2) and performed QTL analysis for stem rust resistance with the graph‐based reference. Based on this graph‐based reference, we identified two significant QTL for stem rust resistance, and the same two QTL were also found by the QTL analysis with a single reference (Rabiosa v2, Figure 5c; Table 2). This suggests that the graph‐based reference together with the current pangenomic tools, such as the PGGB (Garrison et al., 2023), haplotype‐aware read mapper (Giraffe, Sirén et al., 2021), and the toolkit to manipulate the graph‐based reference (vg toolkit, Garrison et al., 2018), can be reliably applied to highly heterozygous *Lolium* genomes. However, several challenges with the pangenomic approach should be noted. First, due to the high sequence variation between haplotypes, haplomes are difficult to align, and a more suitable WGA tool is needed. For example, instead of using the standard aligner wfmash in PGGB, we used AnchorWave (Song et al., 2022), which is a synteny‐based aligner for pair‐wise WGA as it is specifically designed for highly heterozygous plant genomes. However, even with AnchorWave, we found that the two haplotypes were not well aligned, leaving the graph‐based reference 1.42 Gb larger than the total size of the pseudo‐chromosomes of Rabiosa v2 (3.66 vs. 2.24 Gb). In contrast, for a pangenome of human built from 90 haplotypes with PGGB, the size of the graph was only 5 Gb, adding just 2 Gb more sequence variations from 90 haplotypes to the haploid genome (genome size 3 Gb, Liao et al., 2023). Second, although QTL analysis worked with the graph‐based reference, we found that the QTL detected on LG 7 with the graph‐based reference showed a lower LOD score compared to the same QTL detected with the single reference. However, this does not necessarily indicate that the QTL analysis with the graph‐based reference performed worse than QTL analysis with the single reference. A possible reason for the lower LOD score with the graph‐based reference could be that fewer SNPs were genotyped in the QTL region with the graph‐based reference due to the difficulty of aligning the two divergent haplotypes (Figure 5c). To improve QTL analysis using the graph‐based reference, small INDELs and large SVs present in the graph‐based reference need to be considered in addition to SNPs (Vaughn et al., 2022) as SNPs may not be called or genotyped if the two haplotypes could not be aligned.

In conclusion, the chromosome‐level unphased haploid assembly (Rabiosa v2) and the two phased haplotype assemblies (Rabiosa h1 and h2) that we have generated provide invaluable genomic resources for the forage and turf grass research and breeding community. They will serve as references for Italian ryegrass for genomic applications such as reference‐assisted scaffolding, reference‐based phasing, QTL analysis, and genome‐wide association studies. The two QTL linked to stem rust resistance were found with the graph‐based reference, and the comparison between the QTL analyses done with the graph‐based reference and the single reference emphasizes the potential of graph‐based reference and pangenomic tools for future research in forage grasses and other highly heterozygous plant species. While the value of such graph‐based references and pangenomic methods may not be fully exploited with the genomic resources presented here, it will increase in the future as more and more complex genomes are becoming available and the community's needs will develop beyond single reference sequences.

## AUTHOR CONTRIBUTIONS

**Yutang Chen**: Data curation; formal analysis; investigation; methodology; visualization; writing—original draft; writing—review and editing. **Jenny Kiesbauer**: Formal analysis; investigation; methodology; writing—review and editing. **Dario Copetti**: Data curation; methodology; supervision; writing—review and editing. **Daniel Frei**: Data curation; methodology; writing—review and editing. **Jürg E. Frey**: Data curation; methodology; writing—review and editing. **Christoph Grieder**: Methodology; resources; writing—review and editing. **Roland Kölliker**: Formal analysis; methodology; supervision; writing—review and editing. **Bruno Studer**: Conceptualization; funding acquisition; supervision; writing—review and editing.

## CONFLICT OF INTEREST STATEMENT

The authors declare no conflicts of interest.

## Supporting information



Figure S1. Consensus genetic linkage maps used for scaffolding Rabiosa v1, as output by Lep‐MAP3 (Rastas, 2017).
Figure S2. Gene‐based synteny between Rabiosa and other Pooideae genomes.
Figure S3. Pair‐wise gene‐based synteny between Rabiosa v2, Rabiosa h1, and h2.
Figure S4. K‐mer profile of the Rabiosa diploid genome generated using GenomeScope2 with k‐mers from whole‐genome sequencing short reads.
Figure S5. Sequence identity between Rabiosa h1 and Rabiosa h2 for the chromosomes Chr2 to Chr7 (the comparison between the two haplotypes of Chr1 is given in the main text).
Figure S6. Histogram of the phenotypic distribution of resistance to stem rust based on the best linear unbiased estimators (BLUEs).
Figure S7. QTL analysis for resistance to stem rust using the genetic linkage map of Sikem.
Figure S8. Reference bias affects reference‐based phasing.

Table S1: Assembly statistics of Rabiosa
Table S2: Statistics of phase blocks
Table S3: Statistics of binned reads from reference‐based phasing
Table S4: BUSCO (v4.1.4) assessment results of genome assemblies
Table S5: Transposable elements (TE) annotation results
Table S6: Number of variants for calculating sequence variation between the two haplotypes of Rabiosa

## Data Availability

The unphased haploid genome assembly Rabiosa v2 is publicly available on NCBI under the accession: JAUUTY000000000. The two haplotype assemblies are publicly available on NCBI under the accession: JBHEQO000000000. The transcripts used in our haplotig purge pipeline generated previously by Copetti et al. (2021), together with the genome annotation results including the GFF files and the functional annotation results, are available at https://doi.org/10.5281/zenodo.13641219. All genetic linkage maps generated in this work together with the stem rust resistance scores (BLUEs) used for QTL analysis are available at https://doi.org/10.5281/zenodo.15009016. All sequencing data (ONT data, WGS short reads, Hi‐C data, and GBS data) generated in this work have been submitted to NCBI under BioProject accession: PRJNA990649. The RNA‐Seq data generated previously by Copetti et al. (2021) are available on NCBI with the BioProject accession: PRJNA1156394. Code used for bioinformatic analyses in this work can be found at GitHub, https://github.com/Yutang‐ETH/Rabiosa.

## References

[tpg270079-bib-0001] Alonge, M. , Lebeigle, L. , Kirsche, M. , Jenike, K. , Ou, S. , Aganezov, S. , Wang, X. , Lippman, Z. B. , Schatz, M. C. , & Soyk, S. (2022). Automated assembly scaffolding using RagTag elevates a new tomato system for high‐throughput genome editing. Genome Biology, 23, Article 258. 10.1186/s13059-022-02823-7 36522651 PMC9753292

[tpg270079-bib-0002] Altschul, S. F. , Gish, W. , Miller, W. , Myers, E. W. , & Lipman, D. J. (1990). Basic local alignment search tool. Journal of Molecular Biology, 215, 403–410. 10.1016/S0022-2836(05)80360-2 2231712

[tpg270079-bib-0003] Andrews, S. (2015). FastQC . Babraham Bioinformatics. https://www.bioinformatics.babraham.ac.uk/projects/fastqc/

[tpg270079-bib-0004] Bao, Z. , Li, C. , Li, G. , Wang, P. , Peng, Z. , Cheng, L. , Li, H. , Zhang, Z. , Li, Y. , Huang, W. U. , Ye, M. , Dong, D. , Cheng, Z. , Vanderzaag, P. , Jacobsen, E. , Bachem, C. W. B. , Dong, S. , Zhang, C. , Huang, S. , & Zhou, Q. (2022). Genome architecture and tetrasomic inheritance of autotetraploid potato. Molecular Plant, 15, 1211–1226. 10.1016/j.molp.2022.06.009 35733345

[tpg270079-bib-0090] Bateman, A. , Martin, M.‐J. , Orchard, S. , Magrane, M. , Agivetova, R. , Ahmad, S. , Alpi, E. , Bowler‐Barnett, E. H. , Britto, R. , Bursteinas, B. , Bye‐A‐Jee, H. , Coetzee, R. , Cukura, A. , Da Silva, A. , Denny, P. , Dogan, T. , Ebenezer, T. , Fan, J. , … Teodoro, D. , The UniProt Consortium . (2021). UniProt: The universal protein knowledgebase in 2021. Nucleic Acids Research, 49, D480–D489. 10.1093/nar/gkaa1100 33237286 PMC7778908

[tpg270079-bib-0005] Bates, D. , Mächler, M. , Bolker, B. , & Walker, S. (2015). Fitting linear mixed‐effects models using lme4. Journal of Statistical Software, 67, 1–48. 10.18637/jss.v067.i01

[tpg270079-bib-0006] Begheyn, R. F. , Yates, S. A. , Sykes, T. , & Studer, B. (2018). Genetic loci governing androgenic capacity in perennial ryegrass (*Lolium perenne* L.). G3 Genes|Genomes|Genetics, 8, 1897–1908. 10.1534/g3.117.300550 29626084 PMC5982819

[tpg270079-bib-0007] Bolger, A. M. , Lohse, M. , & Usadel, B. (2014). Trimmomatic: A flexible trimmer for Illumina sequence data. Bioinformatics, 30, 2114–2120. 10.1093/bioinformatics/btu170 24695404 PMC4103590

[tpg270079-bib-0008] Boller, B. , Posselt, U. K. , & Veronesi, F. (Eds.). (2010). Fodder crops and amenity grasses (Vol. 212). Springer. 10.1007/978-1-4419-0760-8

[tpg270079-bib-0009] Broman, K. W. , Wu, H. , Sen, Ś. , & Churchill, G. A. (2003). R/qtl: QTL mapping in experimental crosses. Bioinformatics, 19, 889–890. 10.1093/bioinformatics/btg112 12724300

[tpg270079-bib-0010] Brůna, T. , Hoff, K. J. , Lomsadze, A. , Stanke, M. , & Borodovsky, M. (2021). BRAKER2: Automatic eukaryotic genome annotation with GeneMark‐EP+ and AUGUSTUS supported by a protein database. NAR Genomics and Bioinformatics, 3, lqaa108. 10.1093/nargab/lqaa108 33575650 PMC7787252

[tpg270079-bib-0011] Brunharo, C. A. , Short, A. W. , Bobadilla, L. K. , & Streisfeld, M. A. (2025). The genome of *Lolium multiflorum* reveals the genetic architecture of paraquat resistance. Molecular Ecology, 34, e17775. 10.1111/mec.17775 40285737 PMC12051776

[tpg270079-bib-0012] Buchfink, B. , Xie, C. , & Huson, D. H. (2015). Fast and sensitive protein alignment using DIAMOND. Nature Methods, 12, 59–60. 10.1038/nmeth.3176 25402007

[tpg270079-bib-0013] Campbell, M. S. , Law, M. , Holt, C. , Stein, J. C. , Moghe, G. D. , Hufnagel, D. E. , Lei, J. , Achawanantakun, R. , Jiao, D. , Lawrence, C. J. , Ware, D. , Shiu, S.‐H. , Childs, K. L. , Sun, Y. , Jiang, N. , & Yandell, M. (2014). MAKER‐P: A tool kit for the rapid creation, management, and quality control of plant genome annotations. Plant Physiology, 164, 513–524. 10.1104/pp.113.230144 24306534 PMC3912085

[tpg270079-bib-0014] Chen, J. , Liu, Y. , Liu, M. , Guo, W. , Wang, Y. , He, Q. , Chen, W. , Liao, Y. , Zhang, W. , Gao, Y. , Dong, K. , Ren, R. , Yang, T. , Zhang, L. , Qi, M. , Li, Z. , Zhao, M. , Wang, H. , Wang, J. , … Diao, X. (2023). Pangenome analysis reveals genomic variations associated with domestication traits in broomcorn millet. Nature Genetics, 55, 2243–2254. 10.1038/s41588-023-01571-z 38036791 PMC10703678

[tpg270079-bib-0015] Chen, S. , Zhou, Y. , Chen, Y. , & Gu, J. (2018). fastp: An ultra‐fast all‐in‐one FASTQ preprocessor. Bioinformatics, 34, i884–i890. 10.1093/bioinformatics/bty560 30423086 PMC6129281

[tpg270079-bib-0016] Chen, Y. , Kölliker, R. , Mascher, M. , Copetti, D. , Himmelbach, A. , Stein, N. , & Studer, B. (2024). An improved chromosome‐level genome assembly of perennial ryegrass (*Lolium perenne* L.). Gigabyte, 2024, 1–11. 10.46471/gigabyte.112 PMC10940895 38496214

[tpg270079-bib-0017] Cheng, H. , Concepcion, G. T. , Feng, X. , Zhang, H. , & Li, H. (2021). Haplotype‐resolved de novo assembly using phased assembly graphs with hifiasm. Nature Methods, 18, 170–175. 10.1038/s41592-020-01056-5 33526886 PMC7961889

[tpg270079-bib-0018] Chin, C.‐S. , Peluso, P. , Sedlazeck, F. J. , Nattestad, M. , Concepcion, G. T. , Clum, A. , Dunn, C. , O'Malley, R. , Figueroa‐Balderas, R. , Morales‐Cruz, A. , Cramer, G. R. , Delledonne, M. , Luo, C. , Ecker, J. R. , Cantu, D. , Rank, D. R. , & Schatz, M. C. (2016). Phased diploid genome assembly with single‐molecule real‐time sequencing. Nature Methods, 13, 1050–1054. 10.1038/nmeth.4035 27749838 PMC5503144

[tpg270079-bib-0019] Cochetel, N. , Minio, A. , Guarracino, A. , Garcia, J. F. , Figueroa‐Balderas, R. , Massonnet, M. , Kasuga, T. , Londo, J. P. , Garrison, E. , Gaut, B. S. , & Cantu, D. (2023). A super‐pangenome of the North American wild grape species. Genome Biology, 24, Article 290. 10.1186/s13059-023-03133-2 38111050 PMC10729490

[tpg270079-bib-0020] Copetti, D. , Yates, S. A. , Vogt, M. M. , Russo, G. , Grieder, C. , Kölliker, R. , & Studer, B. (2021). Evidence for high intergenic sequence variation in heterozygous Italian ryegrass (Lolium multiflorum Lam.) genome revealed by a high‐quality draft diploid genome assembly. BioRxiv. 10.1101/2021.05.05.442707

[tpg270079-bib-0021] Danecek, P. , Bonfield, J. K. , Liddle, J. , Marshall, J. , Ohan, V. , Pollard, M. O. , Whitwham, A. , Keane, T. , Mccarthy, S. A. , Davies, R. M. , & Li, H. (2021). Twelve years of SAMtools and BCFtools. GigaScience, 10, giab008. 10.1093/gigascience/giab008 33590861 PMC7931819

[tpg270079-bib-0022] Dudchenko, O. , Batra, S. S. , Omer, A. D. , Nyquist, S. K. , Hoeger, M. , Durand, N. C. , Shamim, M. S. , Machol, I. , Lander, E. S. , Aiden, A. P. , & Aiden, E. L. (2017). De novo assembly of the *Aedes aegypti* genome using Hi‐C yields chromosome‐length scaffolds. Science, 356, 92–95. 10.1126/science.aal3327 28336562 PMC5635820

[tpg270079-bib-0023] Durand, N. C. , Robinson, J. T. , Shamim, M. S. , Machol, I. , Mesirov, J. P. , Lander, E. S. , & Aiden, E. L. (2016). Juicebox provides a visualization system for Hi‐C contact maps with unlimited zoom. Cell Systems, 3, 99–101. 10.1016/j.cels.2015.07.012 27467250 PMC5596920

[tpg270079-bib-0024] Durand, N. C. , Shamim, M. S. , Machol, I. , Rao, S. S. P. , Huntley, M. H. , Lander, E. S. , & Aiden, E. L. (2016). Juicer provides a one‐click system for analyzing loop‐resolution Hi‐C experiments. Cell Systems, 3, 95–98. 10.1016/j.cels.2016.07.002 27467249 PMC5846465

[tpg270079-bib-0025] Edge, P. , Bafna, V. , & Bansal, V. (2016). HapCUT2: Robust and accurate haplotype assembly for diverse sequencing technologies. Genome Research, 27, 801–812. 10.1101/gr.213462.116 27940952 PMC5411775

[tpg270079-bib-0026] Flynn, J. M. , Hubley, R. , Goubert, C. , Rosen, J. , Clark, A. G. , Feschotte, C. , & Smit, A. F. (2020). RepeatModeler2 for automated genomic discovery of transposable element families. Proceedings of the National Academy of Sciences of the USA, 117, 9451–9457. 10.1073/pnas.1921046117 32300014 PMC7196820

[tpg270079-bib-0027] Frei, D. , Veekman, E. , Grogg, D. , Stoffel‐Studer, I. , Morishima, A. , Shimizu‐Inatsugi, R. , Yates, S. , Shimizu, K. K. , Frey, J. E. , Studer, B. , & Copetti, D. (2021). Ultralong Oxford Nanopore reads enable the development of a reference‐grade perennial ryegrass genome assembly. Genome Biology and Evolution, 13, evab159. 10.1093/gbe/evab159 34247248 PMC8358221

[tpg270079-bib-0028] Garg, S. , Fungtammasan, A. , Carroll, A. , Chou, M. , Schmitt, A. , Zhou, X. , Mac, S. , Peluso, P. , Hatas, E. , Ghurye, J. , Maguire, J. , Mahmoud, M. , Cheng, H. , Heller, D. , Zook, J. M. , Moemke, T. , Marschall, T. , Sedlazeck, F. J. , Aach, J. , … Li, H. (2021). Chromosome‐scale, haplotype‐resolved assembly of human genomes. Nature Biotechnology, 39, 309–312. 10.1038/s41587-020-0711-0 PMC7954703 33288905

[tpg270079-bib-0029] Garrison, E. , Guarracino, A. , Heumos, S. , Villani, F. , Bao, Z. , Tattini, L. , Hagmann, J. , Vorbrugg, S. , Marco‐Sola, S. , Kubica, C. , Ashbrook, D. G. , Thorell, K. , Rusholme‐Pilcher, R. L. , Liti, G. , Rudbeck, E. , Golicz, A. A. , Nahnsen, S. , Yang, Z. , Mwaniki, M. N. , … Prins, P. (2023). Building pangenome graphs. BioRxiv. 10.1038/s41592-024-02430-3 39433878

[tpg270079-bib-0030] Garrison, E. , Sirén, J. , Novak, A. M. , Hickey, G. , Eizenga, J. M. , Dawson, E. T. , Jones, W. , Garg, S. , Markello, C. , Lin, M. F. , Paten, B. , & Durbin, R. (2018). Variation graph toolkit improves read mapping by representing genetic variation in the reference. Nature Biotechnology, 36, 875–879. 10.1038/nbt.4227 PMC6126949 30125266

[tpg270079-bib-0031] Ghurye, J. , Rhie, A. , Walenz, B. P. , Schmitt, A. , Selvaraj, S. , Pop, M. , Phillippy, A. M. , & Koren, S. (2019). Integrating Hi‐C links with assembly graphs for chromosome‐scale assembly. PLoS Computational Biology, 15, e1007273. 10.1371/journal.pcbi.1007273 31433799 PMC6719893

[tpg270079-bib-0032] Goel, M. , Sun, H. , Jiao, W.‐B. , & Schneeberger, K. (2019). SyRI: Finding genomic rearrangements and local sequence differences from whole‐genome assemblies. Genome Biology, 20, Article 277. 10.1186/s13059-019-1911-0 31842948 PMC6913012

[tpg270079-bib-0033] Gremme, G. , Brendel, V. , Sparks, M. E. , & Kurtz, S. (2005). Engineering a software tool for gene structure prediction in higher organisms. Information and Software Technology, 47, 965–978. 10.1016/j.infsof.2005.09.005

[tpg270079-bib-0034] Gremme, G. , Steinbiss, S. , & Kurtz, S. (2013). GenomeTools: A comprehensive software library for efficient processing of structured genome annotations. IEEE/ACM Transactions on Computational Biology and Bioinformatics, 10, 645–656. 10.1109/TCBB.2013.68 24091398

[tpg270079-bib-0035] Guan, D. , McCarthy, S. A. , Wood, J. , Howe, K. , Wang, Y. , & Durbin, R. (2020). Identifying and removing haplotypic duplication in primary genome assemblies. Bioinformatics, 36, 2896–2898. 10.1093/bioinformatics/btaa025 31971576 PMC7203741

[tpg270079-bib-0036] Gui, S. , Wei, W. , Jiang, C. , Luo, J. , Chen, L. U. , Wu, S. , Li, W. , Wang, Y. , Li, S. , Yang, N. , Li, Q. , Fernie, A. R. , & Yan, J. (2022). A pan‐Zea genome map for enhancing maize improvement. Genome biology, 23, 178. 10.1186/s13059-022-02742-7 35999561 PMC9396798

[tpg270079-bib-0037] Guk, J.‐Y. , Jang, M.‐J. , Choi, J.‐W. , Lee, Y. M. , & Kim, S. (2022). De novo phasing resolves haplotype sequences in complex plant genomes. Plant Biotechnology Journal, 20, 1031–1041. 10.1111/pbi.13815 35332665 PMC9129073

[tpg270079-bib-0038] Haas, B. J. (2018). TransDecoder . GitHub. https://github.com/TransDecoder/TransDecoder

[tpg270079-bib-0039] Haas, B. J. , Salzberg, S. L. , Zhu, W. , Pertea, M. , Allen, J. E. , Orvis, J. , White, O. , Buell, C. R. , & Wortman, J. R. (2008). Automated eukaryotic gene structure annotation using EVidenceModeler and the Program to Assemble Spliced Alignments. Genome Biology, 9, Article R7. 10.1186/gb-2008-9-1-r7 18190707 PMC2395244

[tpg270079-bib-0040] Hayes, B. J. , Cogan, N. O. I. , Pembleton, L. W. , Goddard, M. E. , Wang, J. , Spangenberg, G. C. , & Forster, J. W. (2013). Prospects for genomic selection in forage plant species. Plant Breeding, 132, 133–143. 10.1111/pbr.12037

[tpg270079-bib-0041] He, Q. , Tang, S. , Zhi, H. , Chen, J. , Zhang, J. , Liang, H. , Alam, O. , Li, H. , Zhang, H. , Xing, L. , Li, X. , Zhang, W. , Wang, H. , Shi, J. , Du, H. , Wu, H. , Wang, L. , Yang, P. , Xing, L. , … Diao, X. (2023). A graph‐based genome and pan‐genome variation of the model plant Setaria. Nature Genetics, 55, 1232–1242. 10.1038/s41588-023-01423-w 37291196 PMC10335933

[tpg270079-bib-0042] Hickey, G. , Heller, D. , Monlong, J. , Sibbesen, J. A. , Sirén, J. , Eizenga, J. , Dawson, E. T. , Garrison, E. , Novak, A. M. , & Paten, B. (2020). Genotyping structural variants in pangenome graphs using the vg toolkit. Genome Biology, 21, Article 35. 10.1186/s13059-020-1941-7 32051000 PMC7017486

[tpg270079-bib-0043] Hu, G. , Feng, J. , Xiang, X. , Wang, J. , Salojärvi, J. , Liu, C. , Wu, Z. , Zhang, J. , Liang, X. , Jiang, Z. , Liu, W. , Ou, L. , Li, J. , Fan, G. , Mai, Y. , Chen, C. , Zhang, X. , Zheng, J. , Zhang, Y. , … Li, J. (2022). Two divergent haplotypes from a highly heterozygous lychee genome suggest independent domestication events for early and late‐maturing cultivars. Nature Genetics, 54, 73–83. 10.1038/s41588-021-00971-3 34980919 PMC8755541

[tpg270079-bib-0044] Jain, C. , Rhie, A. , Hansen, N. F. , Koren, S. , & Phillippy, A. M. (2022). Long‐read mapping to repetitive reference sequences using Winnowmap2. Nature Methods, 19, 705–710. 10.1038/s41592-022-01457-8 35365778 PMC10510034

[tpg270079-bib-0045] Jayakodi, M. , Padmarasu, S. , Haberer, G. , Bonthala, V. S. , Gundlach, H. , Monat, C. , Lux, T. , Kamal, N. , Lang, D. , Himmelbach, A. , Ens, J. , Zhang, X.‐Q. , Angessa, T. T. , Zhou, G. , Tan, C. , Hill, C. , Wang, P. , Schreiber, M. , Boston, L. B. , … Stein, N. (2020). The barley pan‐genome reveals the hidden legacy of mutation breeding. Nature, 588, 284–289. 10.1038/s41586-020-2947-8 33239781 PMC7759462

[tpg270079-bib-0046] Jones, P. , Binns, D. , Chang, H.‐Y. , Fraser, M. , Li, W. , Mcanulla, C. , Mcwilliam, H. , Maslen, J. , Mitchell, A. , Nuka, G. , Pesseat, S. , Quinn, A. F. , Sangrador‐Vegas, A. , Scheremetjew, M. , Yong, S.‐Y. , Lopez, R. , & Hunter, S. (2014). InterProScan 5: Genome‐scale protein function classification. Bioinformatics, 30, 1236–1240. 10.1093/bioinformatics/btu031 24451626 PMC3998142

[tpg270079-bib-0047] Kiełbasa, S. M. , Wan, R. , Sato, K. , Horton, P. , & Frith, M. C. (2011). Adaptive seeds tame genomic sequence comparison. Genome Research, 21, 487–493. 10.1101/gr.113985.110 21209072 PMC3044862

[tpg270079-bib-0048] Kim, D. , Langmead, B. , & Salzberg, S. L. (2015). HISAT: A fast spliced aligner with low memory requirements. Nature Methods, 12, 357–360. 10.1038/nmeth.3317 25751142 PMC4655817

[tpg270079-bib-0049] Knorst, V. , Yates, S. , Byrne, S. , Asp, T. , Widmer, F. , Studer, B. , & Kölliker, R. (2019). First assembly of the gene‐space of *Lolium multiflorum* and comparison to other Poaceae genomes. Grassland Science, 65, 125–134. 10.1111/grs.12225

[tpg270079-bib-0050] Kolmogorov, M. , Yuan, J. , Lin, Y. , & Pevzner, P. A. (2019). Assembly of long, error‐prone reads using repeat graphs. Nature Biotechnology, 37, 540–546. 10.1038/s41587-019-0072-8 30936562

[tpg270079-bib-0051] Koren, S. , Rhie, A. , Walenz, B. P. , Dilthey, A. T. , Bickhart, D. M. , Kingan, S. B. , Hiendleder, S. , Williams, J. L. , Smith, T. P. L. , & Phillippy, A. M. (2018). De novo assembly of haplotype‐resolved genomes with trio binning. Nature Biotechnology, 36, 1174–1182. 10.1038/nbt.4277 PMC6476705 30346939

[tpg270079-bib-0052] Koren, S. , Walenz, B. P. , Berlin, K. , Miller, J. R. , Bergman, N. H. , & Phillippy, A. M. (2017). Canu: Scalable and accurate long‐read assembly via adaptive *k* ‐mer weighting and repeat separation. Genome Research, 27, 722–736. 10.1101/gr.215087.116 28298431 PMC5411767

[tpg270079-bib-0053] Li, H. (2013). Aligning sequence reads, clone sequences and assembly contigs with BWA‐MEM. arXiv. 10.48550/arXiv.1303.3997

[tpg270079-bib-0054] Li, H. (2018). Minimap2: Pairwise alignment for nucleotide sequences. Bioinformatics, 34, 3094–3100. 10.1093/bioinformatics/bty191 29750242 PMC6137996

[tpg270079-bib-0055] Li, H. , & Durbin, R. (2024). Genome assembly in the telomere‐to‐telomere era. Nature Reviews Genetics, 25, 658–670. 10.1038/s41576-024-00718-w 38649458

[tpg270079-bib-0056] Liao, W.‐W. , Asri, M. , Ebler, J. , Doerr, D. , Haukness, M. , Hickey, G. , Lu, S. , Lucas, J. K. , Monlong, J. , Abel, H. J. , Buonaiuto, S. , Chang, X. H. , Cheng, H. , Chu, J. , Colonna, V. , Eizenga, J. M. , Feng, X. , Fischer, C. , Fulton, R. S. , … Paten, B. (2023). A draft human pangenome reference. Nature, 617, 312–324. 10.1038/s41586-023-05896-x 37165242 PMC10172123

[tpg270079-bib-0057] Liu, Y. , Du, H. , Li, P. , Shen, Y. , Peng, H. , Liu, S. , Zhou, G.‐A. , Zhang, H. , Liu, Z. , Shi, M. , Huang, X. , Li, Y. , Zhang, M. , Wang, Z. , Zhu, B. , Han, B. , Liang, C. , & Tian, Z. (2020). Pan‐genome of wild and cultivated soybeans. Cell, 182, 162–176. E13. 10.1016/j.cell.2020.05.023 32553274

[tpg270079-bib-0058] Llamas, B. , Narzisi, G. , Schneider, V. , Audano, P. A. , Biederstedt, E. , Blauvelt, L. , Bradbury, P. , Chang, X. , Chin, C.‐S. , Fungtammasan, A. , Clarke, W. E. , Cleary, A. , Ebler, J. , Eizenga, J. , Sibbesen, J. A. , Markello, C. J. , Garrison, E. , Garg, S. , Hickey, G. , … Busby, B. (2021). A strategy for building and using a human reference pangenome. F1000Research, 8, 1751. 10.12688/f1000research.19630.2 PMC8350888 34386196

[tpg270079-bib-0059] Mak, Q. X. C. , Wick, R. R. , Holt, J. M. , & Wang, J. R. (2023). Polishing de novo nanopore assemblies of bacteria and eukaryotes With FMLRC2. Molecular Biology and Evolution, 40, msad048. 10.1093/molbev/msad048 36869750 PMC10015616

[tpg270079-bib-0060] Manni, M. , Berkeley, M. R. , Seppey, M. , & Zdobnov, E. M. (2021). BUSCO: Assessing genomic data quality and beyond. Current Protocols, 1, e323. 10.1002/cpz1.323 34936221

[tpg270079-bib-0061] Mapleson, D. , Garcia Accinelli, G. , Kettleborough, G. , Wright, J. , & Clavijo, B. J. (2017). KAT: A K‐mer analysis toolkit to quality control NGS datasets and genome assemblies. Bioinformatics, 33, 574–576. 10.1093/bioinformatics/btw663 27797770 PMC5408915

[tpg270079-bib-0062] Martin, M. , Patterson, M. , Garg, S. , Fischer, S. O. , Pisanti, N. , Klau, G. W. , Schöenhuth, A. , & Marschall, T. (2016). WhatsHap: Fast and accurate read‐based phasing. BioRxiv. 10.1101/085050

[tpg270079-bib-0063] Mascher, M. , Wicker, T. , Jenkins, J. , Plott, C. , Lux, T. , Koh, C. S. , Ens, J. , Gundlach, H. , Boston, L. B. , Tulpová, Z. , Holden, S. , Hernández‐Pinzón, I. , Scholz, U. , Mayer, K. F. X. , Spannagl, M. , Pozniak, C. J. , Sharpe, A. G. , Šimková, H. , Moscou, M. J. , … Stein, N. (2021). Long‐read sequence assembly: A technical evaluation in barley. Plant Cell, 33, 1888–1906. 10.1093/plcell/koab077 33710295 PMC8290290

[tpg270079-bib-0064] Mistry, J. , Chuguransky, S. , Williams, L. , Qureshi, M. , Salazar, G. A. , Sonnhammer, E. L. L. , Tosatto, S. C. E. , Paladin, L. , Raj, S. , Richardson, L. J. , Finn, R. D. , & Bateman, A. (2021). Pfam: The protein families database in 2021. Nucleic Acids Research, 49, D412–D419. 10.1093/nar/gkaa913 33125078 PMC7779014

[tpg270079-bib-0065] Monat, C. , Padmarasu, S. , Lux, T. , Wicker, T. , Gundlach, H. , Himmelbach, A. , Ens, J. , Li, C. , Muehlbauer, G. J. , Schulman, A. H. , Waugh, R. , Braumann, I. , Pozniak, C. , Scholz, U. , Mayer, K. F. X. , Spannagl, M. , Stein, N. , & Mascher, M. (2019). TRITEX: Chromosome‐scale sequence assembly of Triticeae genomes with open‐source tools. Genome Biology, 20, Article 284. 10.1186/s13059-019-1899-5 31849336 PMC6918601

[tpg270079-bib-0066] Money, D. , Migicovsky, Z. , Gardner, K. , & Myles, S. (2017). LinkImputeR: User‐guided genotype calling and imputation for non‐model organisms. BMC Genomics, 18, Article 523. 10.1186/s12864-017-3873-5 28693460 PMC5504746

[tpg270079-bib-0067] Nagy, I. , Veeckman, E. , Liu, C. , Bel, M. V. , Vandepoele, K. , Jensen, C. S. , Ruttink, T. , & Asp, T. (2022). Chromosome‐scale assembly and annotation of the perennial ryegrass genome. BMC Genomics, 23, Article 505. 10.1186/s12864-022-08697-0 35831814 PMC9281035

[tpg270079-bib-0068] Najoshi . (2022). Sabre—A barcode demultiplexing and trimming tool for FastQ files . GitHub, Inc. https://github.com/najoshi/sabre

[tpg270079-bib-0069] Paril, J. , Pandey, G. , Barnett, E. M. , Rane, R. V. , Court, L. , Walsh, T. , & Fournier‐Level, A. (2022). Rounding up the annual ryegrass genome: High‐quality reference genome of *Lolium rigidum* . Frontiers in Genetics, 13, 1012694. 10.3389/fgene.2022.1012694 36386808 PMC9664059

[tpg270079-bib-0070] Pedersen, B. S. , & Quinlan, A. R. (2018). Mosdepth: Quick coverage calculation for genomes and exomes. Bioinformatics, 34, 867–868. 10.1093/bioinformatics/btx699 29096012 PMC6030888

[tpg270079-bib-0071] Pertea, G. , & Pertea, M. (2020). GFF Utilities: GffRead and GffCompare. F1000Research, 9, 304. 10.12688/f1000research.23297.1 PMC7222033 32489650

[tpg270079-bib-0072] Pertea, M. , Kim, D. , Pertea, G. M. , Leek, J. T. , & Salzberg, S. L. (2016). Transcript‐level expression analysis of RNA‐seq experiments with HISAT, StringTie and Ballgown. Nature Protocols, 11, 1650–1667. 10.1038/nprot.2016.095 27560171 PMC5032908

[tpg270079-bib-0073] Pertea, M. , Pertea, G. M. , Antonescu, C. M. , Chang, T.‐C. , Mendell, J. T. , & Salzberg, S. L. (2015). StringTie enables improved reconstruction of a transcriptome from RNA‐seq reads. Nature Biotechnology, 33, 290–295. 10.1038/nbt.3122 PMC4643835 25690850

[tpg270079-bib-0074] Pfeifer, M. , Martis, M. , Asp, T. , Mayer, K. F. X. , Lübberstedt, T. , Byrne, S. , Frei, U. , & Studer, B. (2013). The perennial ryegrass genomezipper: Targeted use of genome resources for comparative grass genomics. Plant Physiology, 161, 571–582. 10.1104/pp.112.207282 23184232 PMC3561004

[tpg270079-bib-0075] Porubsky, D. , Ebert, P. , Audano, P. A. , Vollger, M. R. , Harvey, W. T. , Marijon, P. , Ebler, J. , Munson, K. M. , Sorensen, M. , Sulovari, A. , Haukness, M. , Ghareghani, M. , Lansdorp, P. M. , Paten, B. , Devine, S. E. , Sanders, A. D. , Lee, C. , Chaisson, M. J. P. , Korbel, J. O. , … Marschall, T. (2021). Fully phased human genome assembly without parental data using single‐cell strand sequencing and long reads. Nature Biotechnology, 39, 302–308. 10.1038/s41587-020-0719-5 PMC7954704 33288906

[tpg270079-bib-0103] R Core Team . (2023). R: A language and environment for statistical computing . R Foundation for Statistical Computing. https://www.R-project.org/

[tpg270079-bib-0076] Rastas, P. (2017). Lep‐MAP3: Robust linkage mapping even for low‐coverage whole genome sequencing data. Bioinformatics, 33, 3726–3732. 10.1093/bioinformatics/btx494 29036272

[tpg270079-bib-0077] Rautiainen, M. , Nurk, S. , Walenz, B. P. , Logsdon, G. A. , Porubsky, D. , Rhie, A. , Eichler, E. E. , Phillippy, A. M. , & Koren, S. (2023). Telomere‐to‐telomere assembly of diploid chromosomes with Verkko. Nature Biotechnology, 41, 1474–1482. 10.1038/s41587-023-01662-6 PMC10427740 36797493

[tpg270079-bib-0078] Roach, M. J. , Schmidt, S. A. , & Borneman, A. R. (2018). Purge Haplotigs: Allelic contig reassignment for third‐gen diploid genome assemblies. BMC Bioinformatics, 19, Article 460. 10.1186/s12859-018-2485-7 30497373 PMC6267036

[tpg270079-bib-0079] Schlagenhauf, E. , & Wicker, T. (2016). The TREP platform: A curated database of transposable elements . University of Zurich. https://trep‐db.uzh.ch

[tpg270079-bib-0080] Schubiger, F. X. , & Boller, B. (2016). Virulence of crown rust isolates (*Puccinia coronata* f.sp. *lolii*) on genotypes of Italian and perennial ryegrass (*Lolium multiflorum* and *L. perenne*). European Journal of Plant Pathology, 144, 141–154. 10.1007/s10658-015-0758-9

[tpg270079-bib-0081] Searle, S. R. , Speed, F. M. , & Milliken, G. A. (1980). Population marginal means in the linear model: An alternative to least squares means. The American Statistician, 34, 216–221. 10.1080/00031305.1980.10483031

[tpg270079-bib-0082] Sedlazeck, F. J. , Rescheneder, P. , Smolka, M. , Fang, H. , Nattestad, M. , von Haeseler, A. , & Schatz, M. C. (2018). Accurate detection of complex structural variations using single‐molecule sequencing. Nature Methods, 15, 461–468. 10.1038/s41592-018-0001-7 29713083 PMC5990442

[tpg270079-bib-0083] Serra Mari, R. , Schrinner, S. , Finkers, R. , Ziegler, F. M. R. , Arens, P. , Schmidt, M. H.‐W. , Usadel, B. , Klau, G. W. , & Marschall, T. (2024). Haplotype‐resolved assembly of a tetraploid potato genome using long reads and low‐depth offspring data. Genome Biology, 25, Article 26. 10.1186/s13059-023-03160-z 38243222 PMC10797741

[tpg270079-bib-0084] Shang, L. , Li, X. , He, H. , Yuan, Q. , Song, Y. , Wei, Z. , Lin, H. , Hu, M. , Zhao, F. , Zhang, C. , Li, Y. , Gao, H. , Wang, T. , Liu, X. , Zhang, H. , Zhang, Y. , Cao, S. , Yu, X. , Zhang, B. , … Qian, Q. (2022). A super pan‐genomic landscape of rice. Cell Research, 32, 878–896. 10.1038/s41422-022-00685-z 35821092 PMC9525306

[tpg270079-bib-0085] Sirén, J. , Monlong, J. , Chang, X. , Novak, A. M. , Eizenga, J. M. , Markello, C. , Sibbesen, J. A. , Hickey, G. , Chang, P.‐C. , Carroll, A. , Gupta, N. , Gabriel, S. , Blackwell, T. W. , Ratan, A. , Taylor, K. D. , Rich, S. S. , Rotter, J. I. , Haussler, D. , Garrison, E. , & Paten, B. (2021). Pangenomics enables genotyping of known structural variants in 5202 diverse genomes. Science, 374, abg8871. 10.1126/science.abg8871 34914532 PMC9365333

[tpg270079-bib-0086] Smit, A. , Hubley, R. , & Green, P. (2013). RepeatMasker Open‐4.0. 2013–2015 . Institute for Systems Biology. http://www.repeatmasker.org

[tpg270079-bib-0087] Song, B. , Marco‐Sola, S. , Moreto, M. , Johnson, L. , Buckler, E. S. , & Stitzer, M. C. (2022). AnchorWave: Sensitive alignment of genomes with high sequence diversity, extensive structural polymorphism, and whole‐genome duplication. Proceedings of the National Academy of Sciences of the USA, 119, e2113075119. 10.1073/pnas.2113075119 34934012 PMC8740769

[tpg270079-bib-0088] Sun, H. , Jiao, W.‐B. , Krause, K. , Campoy, J. A. , Goel, M. , Folz‐Donahue, K. , Kukat, C. , Huettel, B. , & Schneeberger, K. (2022). Chromosome‐scale and haplotype‐resolved genome assembly of a tetraploid potato cultivar. Nature Genetics, 54, 342–348. 10.1038/s41588-022-01015-0 35241824 PMC8920897

[tpg270079-bib-0089] Tang, H. , Zhang, X. , Miao, C. , Zhang, J. , Ming, R. , Schnable, J. C. , Schnable, P. S. , Lyons, E. , & Lu, J. (2015). ALLMAPS: Robust scaffold ordering based on multiple maps. Genome Biology, 16, Article 3. 10.1186/s13059-014-0573-1 25583564 PMC4305236

[tpg270079-bib-0091] Trapnell, C. , Roberts, A. , Goff, L. , Pertea, G. , Kim, D. , Kelley, D. R. , Pimentel, H. , Salzberg, S. L. , Rinn, J. L. , & Pachter, L. (2012). Differential gene and transcript expression analysis of RNA‐seq experiments with TopHat and Cufflinks. Nature Protocols, 7, 562–578. 10.1038/nprot.2012.016 22383036 PMC3334321

[tpg270079-bib-0092] Vaughn, J. N. , Branham, S. E. , Abernathy, B. , Hulse‐Kemp, A. M. , Rivers, A. R. , Levi, A. , & Wechter, W. P. (2022). Graph‐based pangenomics maximizes genotyping density and reveals structural impacts on fungal resistance in melon. Nature Communications, 13, Article 7897. 10.1038/s41467-022-35621-7 PMC9780226 36550124

[tpg270079-bib-0093] Walkowiak, S. , Gao, L. , Monat, C. , Haberer, G. , Kassa, M. T. , Brinton, J. , Ramirez‐Gonzalez, R. H. , Kolodziej, M. C. , Delorean, E. , Thambugala, D. , Klymiuk, V. , Byrns, B. , Gundlach, H. , Bandi, V. , Siri, J. N. , Nilsen, K. , Aquino, C. , Himmelbach, A. , Copetti, D. , … Pozniak, C. J. (2020). Multiple wheat genomes reveal global variation in modern breeding. Nature, 588, 277–283. 10.1038/s41586-020-2961-x 33239791 PMC7759465

[tpg270079-bib-0094] Wang, Y. , Tang, H. , Debarry, J. D. , Tan, X. , Li, J. , Wang, X. , Lee, T.‐H. , Jin, H. , Marler, B. , Guo, H. , Kissinger, J. C. , & Paterson, A. H. (2012). MCScanX: A toolkit for detection and evolutionary analysis of gene synteny and collinearity. Nucleic Acids Research, 40, e49–e49. 10.1093/nar/gkr1293 22217600 PMC3326336

[tpg270079-bib-0095] Wu, T. D. , & Watanabe, C. K. (2005). GMAP: A genomic mapping and alignment program for mRNA and EST sequences. Bioinformatics, 21, 1859–1875. 10.1093/bioinformatics/bti310 15728110

[tpg270079-bib-0096] Yan, H. , Sun, M. , Zhang, Z. , Jin, Y. , Zhang, A. , Lin, C. , Wu, B. , He, M. , Xu, B. , Wang, J. , Qin, P. , Mendieta, J. P. , Nie, G. , Wang, J. , Jones, C. S. , Feng, G. , Srivastava, R. K. , Zhang, X. , Bombarely, A. , … Huang, L. (2023). Pangenomic analysis identifies structural variation associated with heat tolerance in pearl millet. Nature Genetics, 55, 507–518. 10.1038/s41588-023-01302-4 36864101 PMC10011142

[tpg270079-bib-0097] Zeng, X. , Yi, Z. , Zhang, X. , Du, Y. , Li, Y. , Zhou, Z. , Chen, S. , Zhao, H. , Yang, S. , Wang, Y. , & Chen, G. (2024). Chromosome‐level scaffolding of haplotype‐resolved assemblies using Hi‐C data without reference genomes. Nature Plants, 10, 1184–1200. 10.1038/s41477-024-01755-3 39103456

[tpg270079-bib-0098] Zhang, X. , Chen, S. , Shi, L. , Gong, D. , Zhang, S. , Zhao, Q. , Zhan, D. , Vasseur, L. , Wang, Y. , Yu, J. , Liao, Z. , Xu, X. , Qi, R. , Wang, W. , Ma, Y. , Wang, P. , Ye, N. , Ma, D. , Shi, Y. , … You, M. (2021). Haplotype‐resolved genome assembly provides insights into evolutionary history of the tea plant *Camellia sinensis* . Nature Genetics, 53, 1250–1259. 10.1038/s41588-021-00895-y 34267370 PMC8346365

[tpg270079-bib-0099] Zhou, Q. , Tang, D. , Huang, W. U. , Yang, Z. , Zhang, Y. , Hamilton, J. P. , Visser, R. G. F. , Bachem, C. W. B. , Robin Buell, C. , Zhang, Z. , Zhang, C. , & Huang, S. (2020). Haplotype‐resolved genome analyses of a heterozygous diploid potato. Nature Genetics, 52, 1018–1023. 10.1038/s41588-020-0699-x 32989320 PMC7527274

[tpg270079-bib-0100] Zhou, Y. , Zhang, Z. , Bao, Z. , Li, H. , Lyu, Y. , Zan, Y. , Wu, Y. , Cheng, L. , Fang, Y. , Wu, K. , Zhang, J. , Lyu, H. , Lin, T. , Gao, Q. , Saha, S. , Mueller, L. , Fei, Z. , Städler, T. , Xu, S. , … Huang, S. (2022). Graph pangenome captures missing heritability and empowers tomato breeding. Nature, 606, 527–534. 10.1038/s41586-022-04808-9 35676474 PMC9200638

[tpg270079-bib-0101] Zimin, A. V. , & Salzberg, S. L. (2020). The genome polishing tool POLCA makes fast and accurate corrections in genome assemblies. PloS Computational Biology, 16, e1007981. 10.1371/journal.pcbi.1007981 32589667 PMC7347232

[tpg270079-bib-0102] Zwyrtková, J. , Němečková, A. , Čížková, J. , Holušová, K. , Kapustová, V. , Svačina, R. , Kopecký, D. , Till, B. J. , Doležel, J. , & Hřibová, E. (2020). Comparative analyses of DNA repeats and identification of a novel Fesreba centromeric element in fescues and ryegrasses. BMC Plant Biology, 20, Article 280. 10.1186/s12870-020-02495-0 32552738 PMC7302162

