# Supplementary material for: Chromosome‐level haplotype‐resolved genome assembly provides insights into the highly heterozygous genome of Italian ryegrass (Lolium multiflorum Lam.)
Source: Plant Genome. 2025 Aug 25;18(3):e70079. doi: 10.1002/tpg2.70079 (PMC12376112; doi:10.1002/tpg2.70079)
Supplement: Supplementary file 1 — Figure S1. Consensus genetic linkage maps used for scaffolding Rabiosa v1, as output by Lep‐MAP3 (Rastas, 2017). Figure S2. Gene‐based synteny between Rabiosa and other Pooideae genomes. Figure S3. Pair‐wise gene‐based synteny between Rabiosa v2, Rabiosa h1, and h2. Figure S4. K‐mer profile of the Rabiosa diploid genome generated using GenomeScope2 with k‐mers from whole‐genome sequencing short reads. Figure S5. Sequence identity between Rabiosa h1 and Rabiosa h2 for the chromosomes Chr2 to Chr7 (the comparison between the two haplotypes of Chr1 is given in the main text). Figure S6. Histogram of the phenotypic distribution of resistance to stem rust based on the best linear unbiased estimators (BLUEs). Figure S7. QTL analysis for resistance to stem rust using the genetic linkage map of Sikem. Figure S8. Reference bias affects reference‐based phasing. [file TPG2-18-e70079-s001.docx]

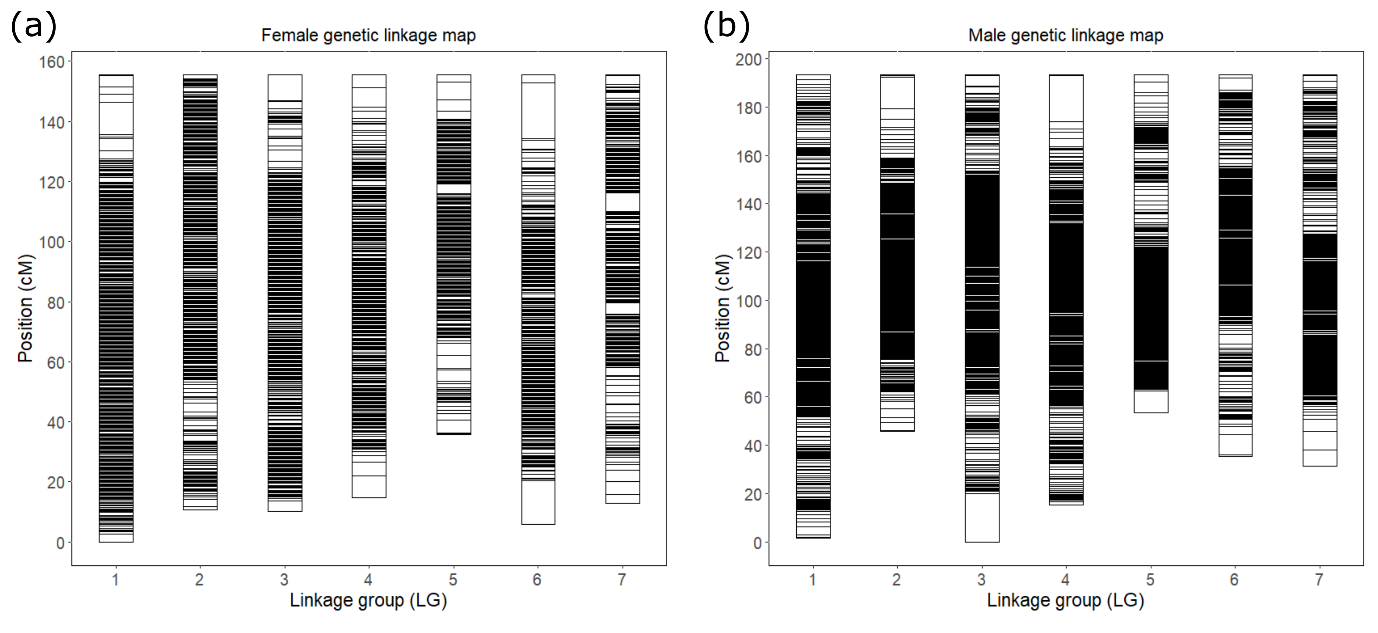


Figure S1. Consensus genetic linkage maps used for scaffolding Rabiosa v1, as output by Lep-MAP3 (Rastas, 2017). Both the female (a) and male (b) consensus genetic linkage maps consist of 26,203 single nucleotide polymorphism (SNP) markers and include informative markers from both parents. Each horizontal black segment within the linkage groups represents one SNP marker.


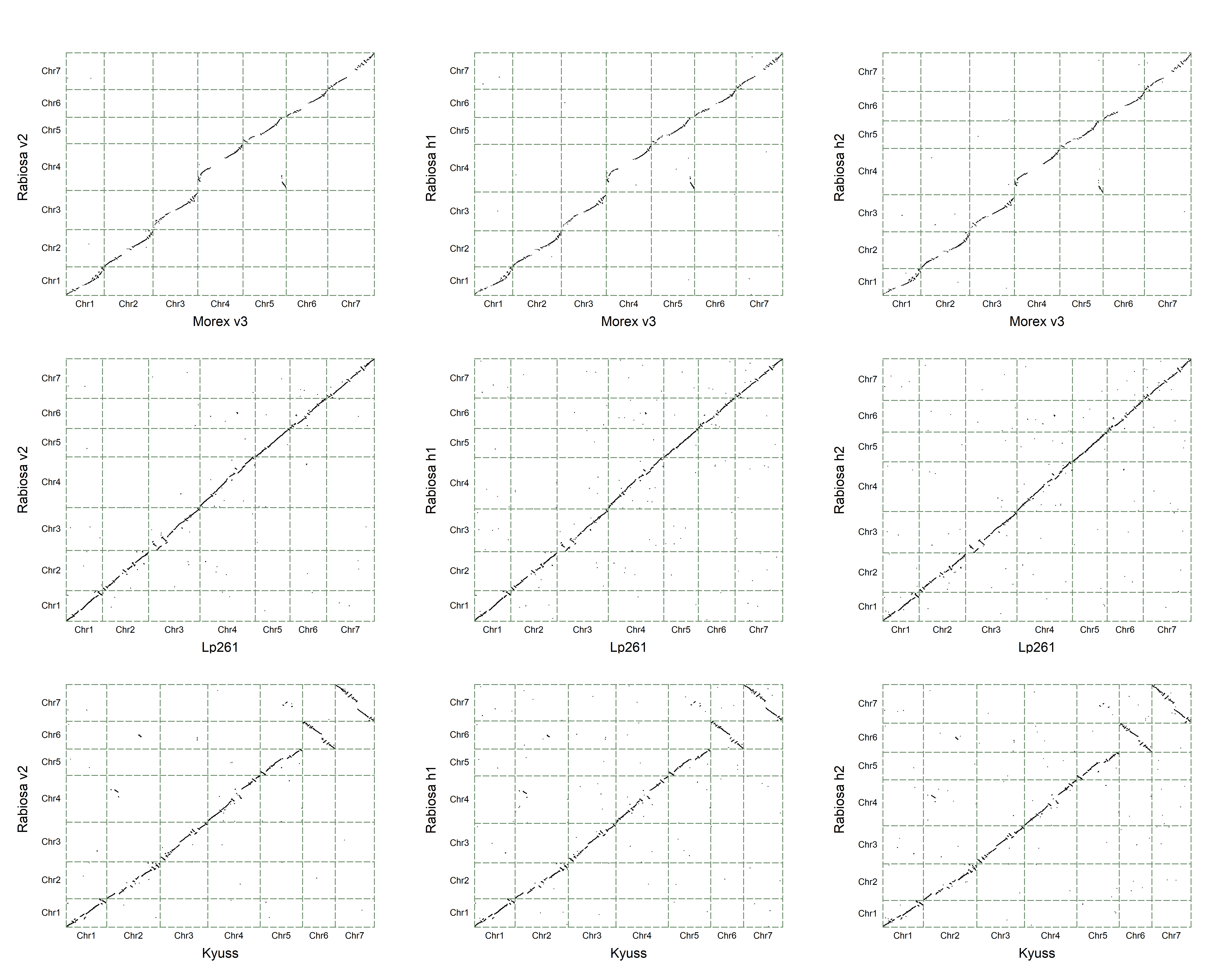


Figure S2. Gene-based synteny between Rabiosa and other Pooideae genomes. Morex v3 (Mascher et al., 2021) is a barley (*Hordeum vulgare*) cultivar. Lp216 (Nagy et al., 2022) is a perennial ryegrass (*Lolium perenne*) genotype. Kyuss (Frei et al., 2021) is a doubled haploid genotype of perennial ryegrass (*L. perenne*).


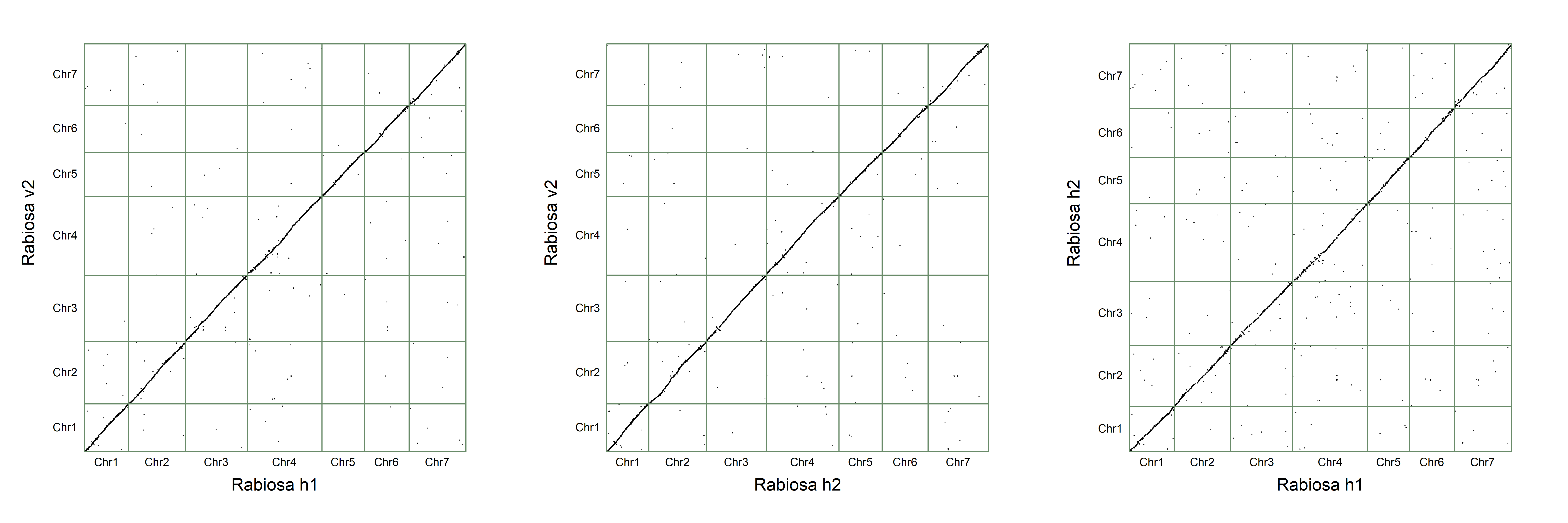


Figure S3. Pair-wise gene-based synteny between Rabiosa v2, Rabiosa h1 and h2.


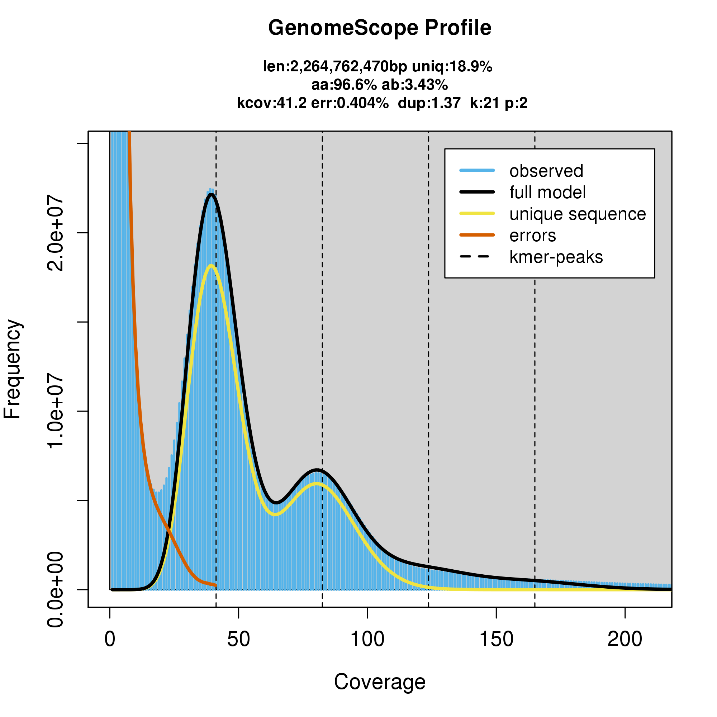


Figure S4. K-mer profile of the Rabiosa diploid genome generated using GenomeScope2 with k-mers from whole-genome sequencing short reads.


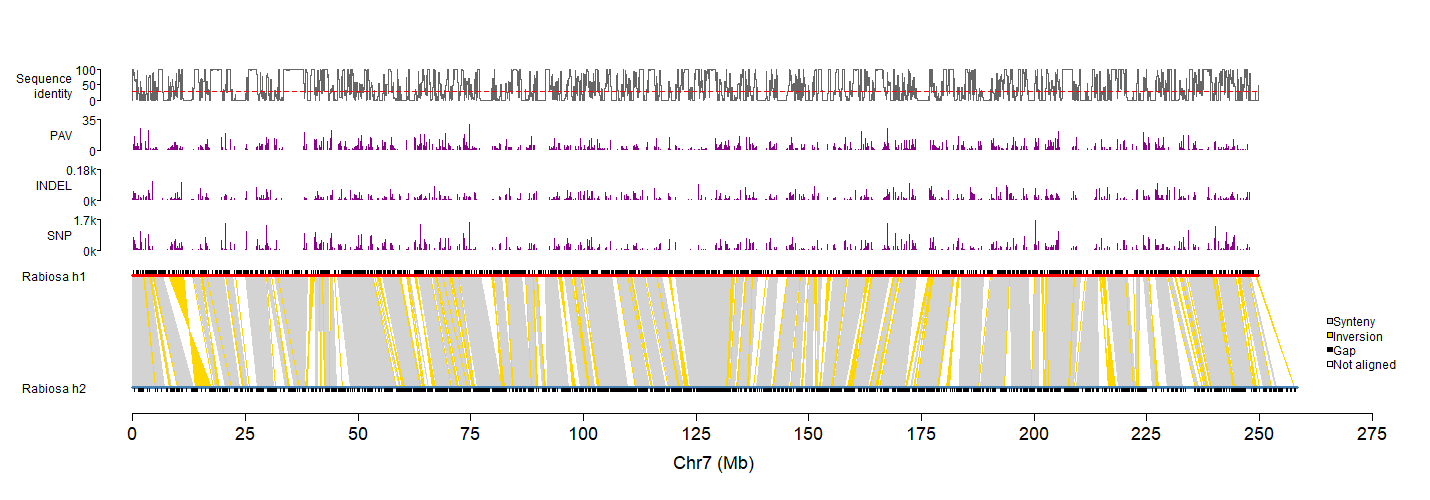

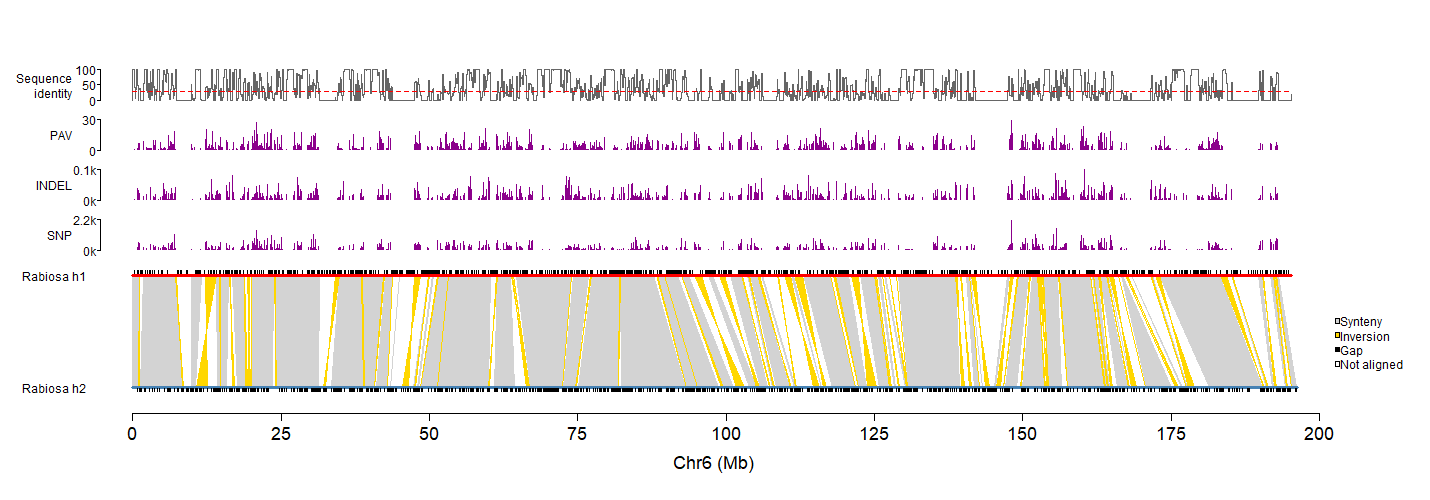

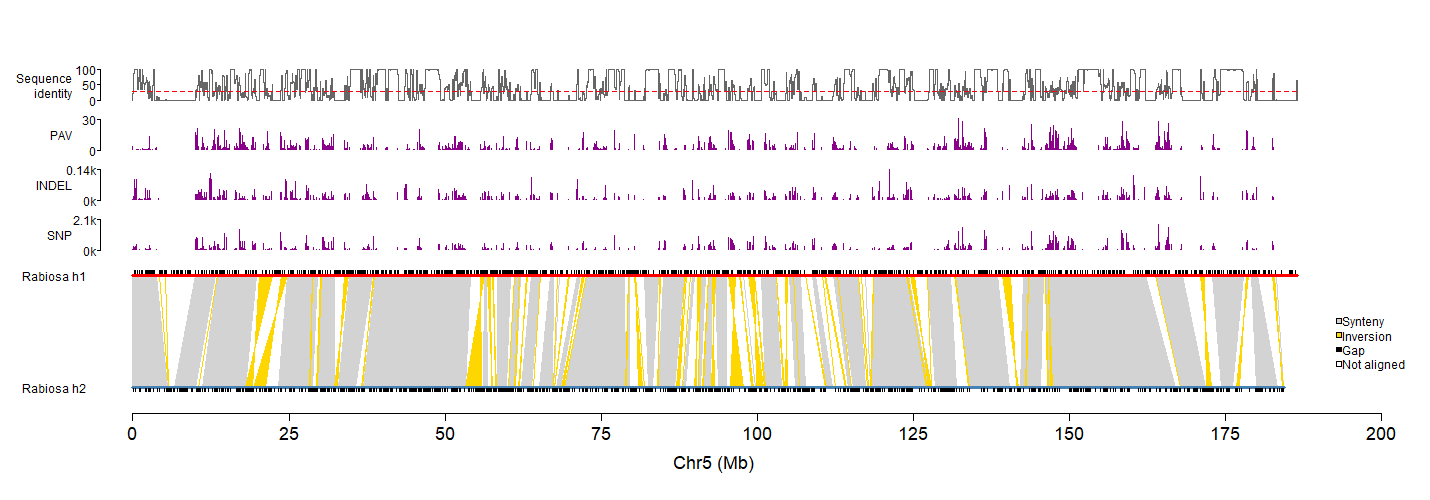

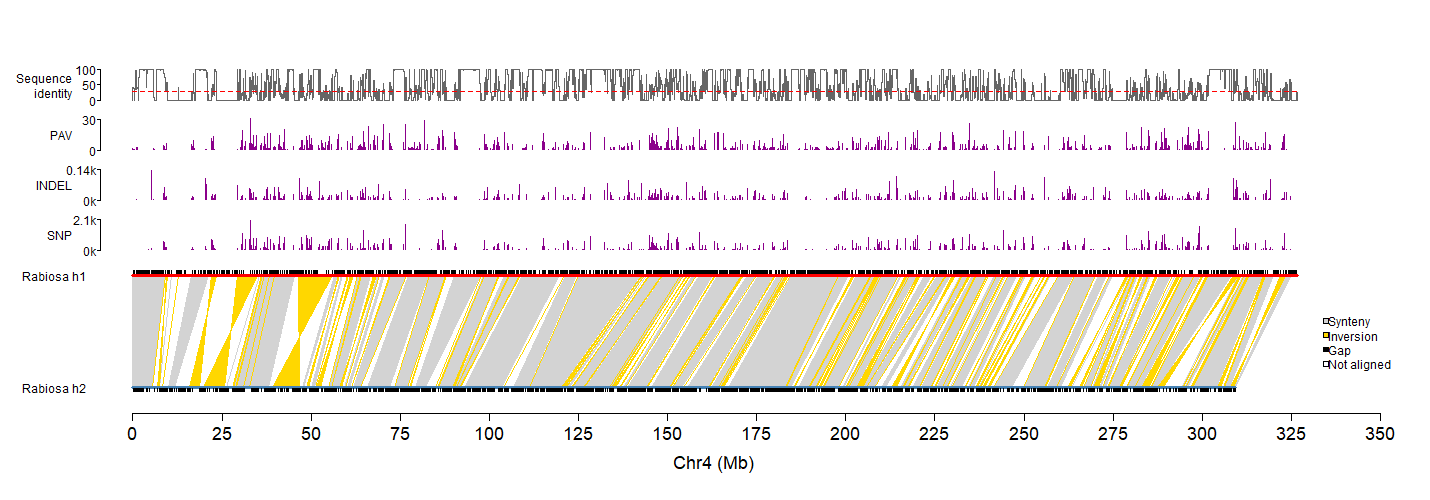

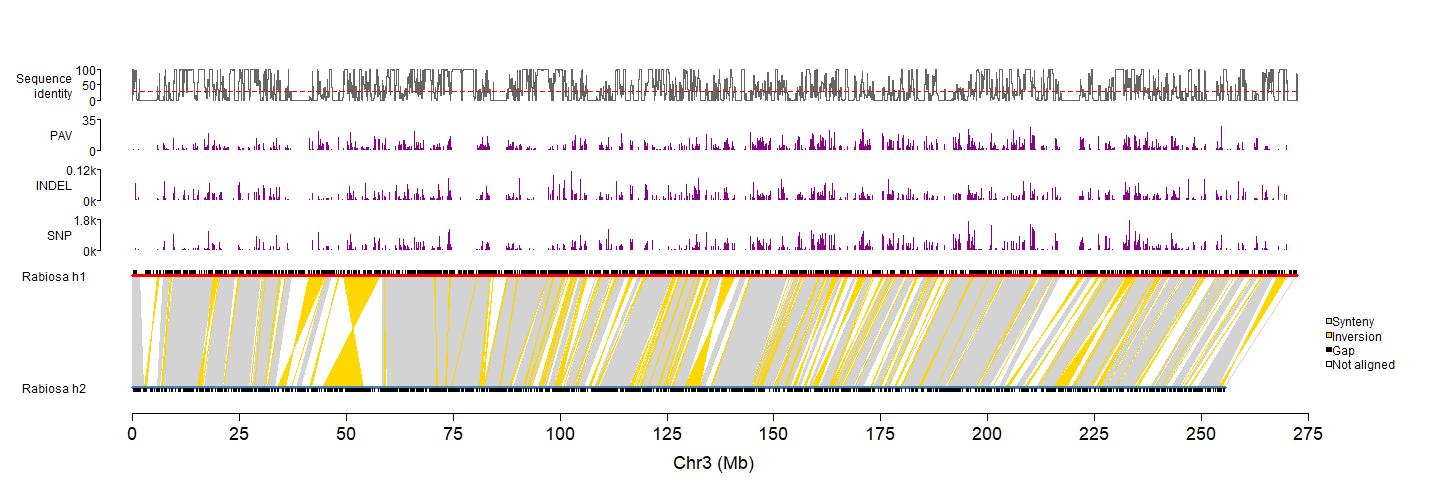

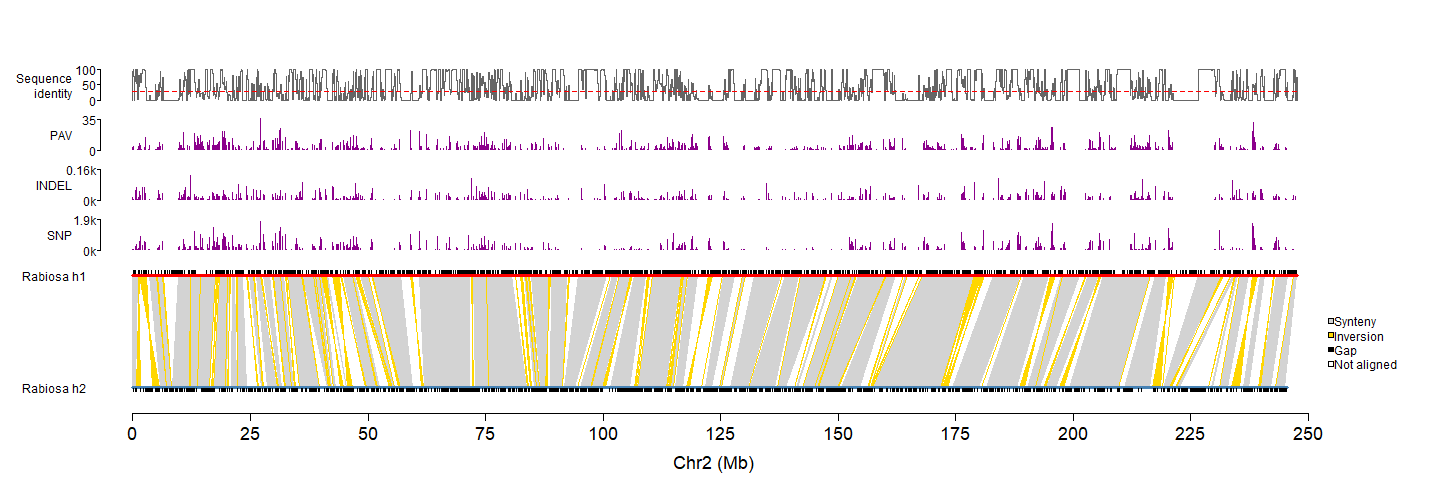


Figure S5. Sequence identity between Rabiosa h1 and Rabiosa h2 for the chromosomes Chr2 to Chr7 (the comparison between the two haplotypes of Chr1 is given in the main text). For each chromosome, the comparison includes (from bottom to up): the length of the pseudo-chromosomes of Rabiosa h1 and h2, the synteny between the two haplotypes and the distribution of gaps (the black ticks), the number of SNPs, INDELs and PAVs found between the two haplotypes (shown per 1 Mb) and the estimated sequence identity per 100 kb between the two haplotypes.


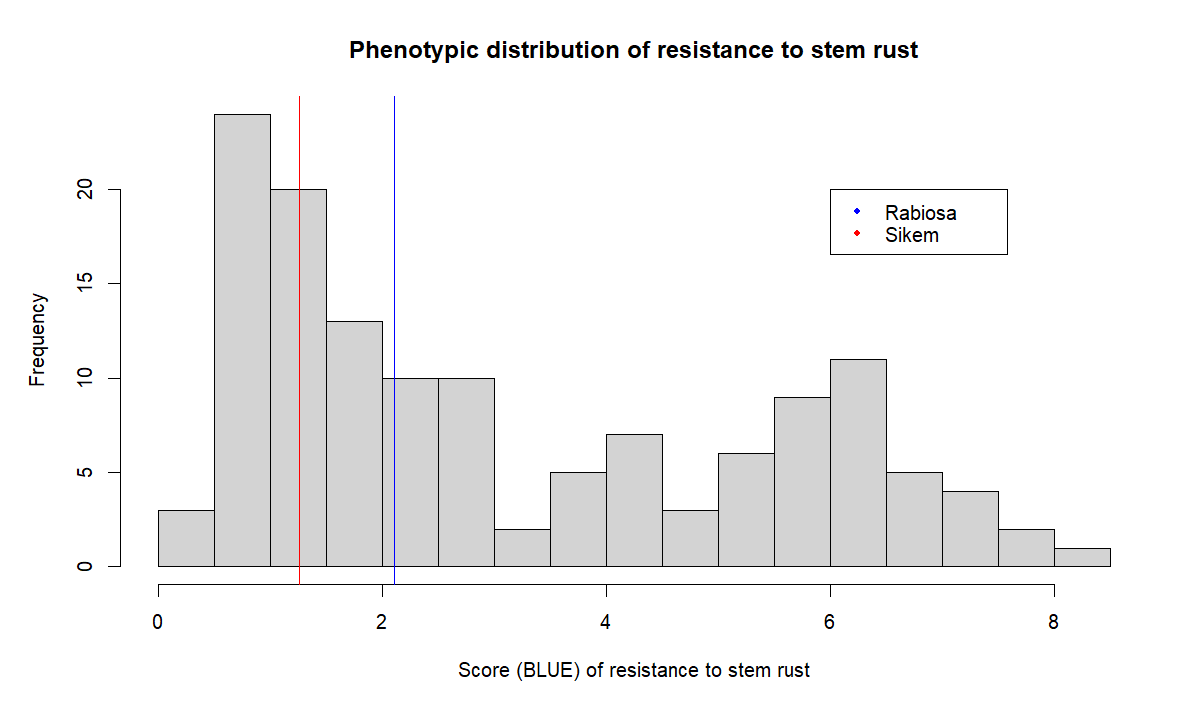


Figure S6. Histogram of the phenotypic distribution of resistance to stem rust based on the best linear unbiased estimators (BLUEs). The BLUEs were calculated based on the occurrence of stem rust (scored using a scale from 1 to 9) for each of the 135 individuals, including 133 F_1_ individuals and the two parents (Rabiosa and Sikem). The BLUEs of the two parents are indicated as blue (Rabiosa) and red (Sikem) lines in the distribution.


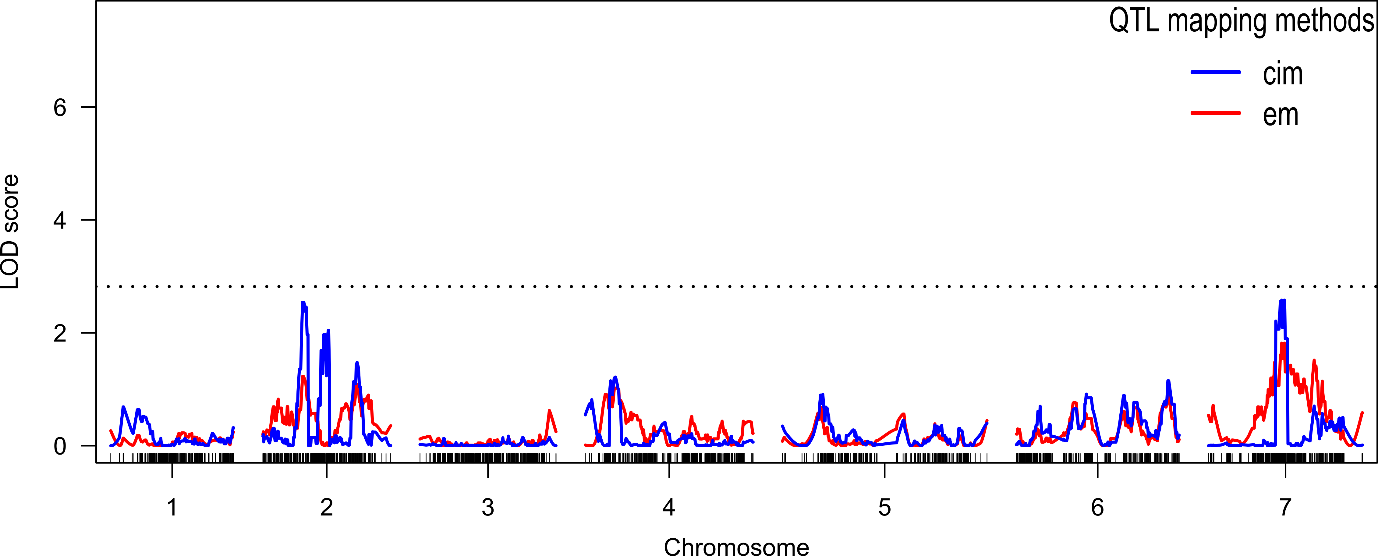


Figure S7. QTL analysis for resistance to stem rust using the genetic linkage map of Sikem. Two interval mapping methods, including simple interval mapping (EM) and composite interval mapping (CIM), were used to conduct QTL analysis, and the results from both methods are shown in the plot as red and blue lines, respectively.


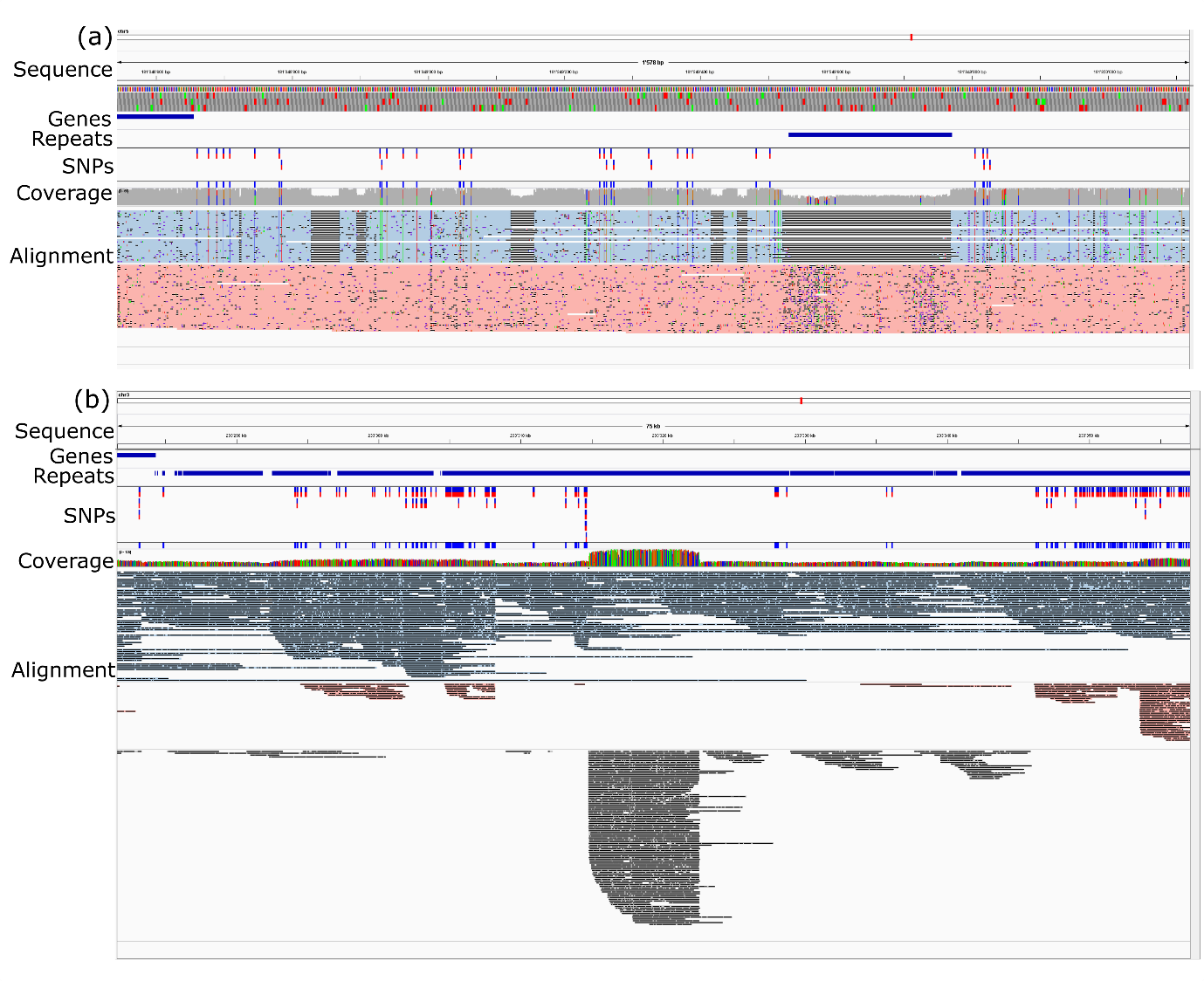


Figure S8. Reference bias affects reference-based phasing. Two IGV screenshots were shown here, and five tracks were shown in each screenshot, indicating, from top to bottom, the position of high confidence genes, the position of repetitive sequences, the phased SNPs, the alignment coverage of the ONT reads and the aligned ONT reads colored by blue, red and gray, representing haplotype 1, haplotype 2 and haplotype unassigned reads, respectively. Screenshot (a) shows the expected reference-based phasing results, where long reads were aligned well to the reference (even though there are large variations) and tagged as different haplotypes. Screenshot (b) shows how reference bias may affect the haplotype assignment in reference-based phasing. In the middle, many reads were left unassigned to any haplotypes (gray). As indicated by the much higher alignment coverage of this middle region compared to neighboring regions, these gray reads (repetitive sequences) are likely wrongly aligned to this region due to reference bias (inaccurate mapping). This suggests that reference bias could cause fewer reads to be assigned to haplotypes. Additionally, it could also be seen that most of the reads aligned in this screenshot are from one haplotype. This is probably because either this repetitive region is only present in one haplotype or reads from the other haplotype could not be accurately aligned to this region. Either case suggests that reference-based phasing may be affected by reference bias.

Methods S1

For genome annotation, protein sequences of the following species were used:

*Brachypodium distachyon*: <https://www.ncbi.nlm.nih.gov/datasets/genome/GCF_000005505.3/>

*Hordeum vulgare*: <https://wheat.pw.usda.gov/GG3/content/morex-v3-files-2021>

*L. perenne,* Kyuss <https://datacommons.cyverse.org/browse/iplant/home/shared/commons_repo/curated/Copetti_Kyuss_assembly_annotation_March_2021>

*L. perenne,* Lp261: <https://ryegrassgenome.ghpc.au.dk/DOWNLOAD/Lolium_2.6.1/v3_transcripts/PROT/>

*Avena sativa*: <https://wheat.pw.usda.gov/GG3/graingenes_downloads/oat-ot3098-pepsico>

*Seccale cereale*: <https://doi.ipk-gatersleben.de/DOI/8afb3971-b5e1-4748-8f0e-1b929ba73248/01369868-8f23-4a21-834f-113b1a9d922d/1/1847940088>).

The transcripts used for genome annotation were collected from following sources: <https://www.ncbi.nlm.nih.gov/geo/query/acc.cgi?acc=GSE144460>

<https://www.ncbi.nlm.nih.gov/geo/query/acc.cgi?acc=GSE78738>

<https://www.ncbi.nlm.nih.gov/geo/query/acc.cgi?acc=GSE141654>

<https://datacommons.cyverse.org/browse/iplant/home/shared/commons_repo/curated/Copetti_Kyuss_assembly_annotation_March_2021>

<https://ryegrassgenome.ghpc.au.dk/>

<https://zenodo.org/record/832654#.Ynovtp0za70>
